# Anti-lipolysis-stimulated lipoprotein receptor monoclonal antibody as a novel therapeutic agent for endometrial cancer

**DOI:** 10.1186/s12885-022-09789-6

**Published:** 2022-06-21

**Authors:** Yoshikazu Nagase, Kosuke Hiramatsu, Masashi Funauchi, Mayu Shiomi, Tatsuo Masuda, Mamoru Kakuda, Satoshi Nakagawa, Ai Miyoshi, Shinya Matsuzaki, Eiji Kobayashi, Toshihiro Kimura, Satoshi Serada, Yutaka Ueda, Tetsuji Naka, Tadashi Kimura

**Affiliations:** 1 Department of Obstetrics and Gynecology, Osaka University Graduate School of Medicine, 2-2 Yamadaoka, Suita, Osaka 565-0871 Japan; 2 Division of Clinical Immunology, Department of Internal Medicine, Iwate Medical University School of Medicine, Iwate, Japan; 3 Institute for Biomedical Sciences Molecular Pathophysiology, Iwate Medical University, Iwate, Japan; 4 Department of Clinical Immunology, Kochi Medical School, Kochi University, Kochi, Japan; 5 Department of Gynecology, Osaka International Cancer Institute, Osaka, Japan

**Keywords:** Antibody therapy, Endometrial cancer, Lipolysis-stimulated lipoprotein receptor, Matrix metalloproteinase, Mitogen-activated protein kinase

## Abstract

**Background:**

Endometrial cancer (EC) is a common gynecologic malignancy and patients with advanced and recurrent EC have a poor prognosis. Although chemotherapy is administered for those patients, the efficacy of current chemotherapy is limited. Therefore, it is necessary to develop novel therapeutic agents for EC. In this study, we focused on lipolysis-stimulated lipoprotein receptor (LSR), a membrane protein highly expressed in EC cells, and developed a chimeric chicken–mouse anti-LSR monoclonal antibody (mAb). This study investigated the antitumor effect of an anti-LSR mAb and the function of LSR in EC.

**Methods:**

We examined the expression of LSR in 228 patients with EC using immunohistochemistry and divided them into two groups: high-LSR (*n* = 153) and low-LSR groups (*n* = 75). We developed a novel anti-LSR mAb and assessed its antitumor activity in an EC cell xenograft mouse model. Pathway enrichment analysis was performed using protein expression data of EC samples. LSR-knockdown EC cell lines (HEC1 and HEC116) were generated by transfected with small interfering RNA and used for assays in vitro.

**Results:**

High expression of LSR was associated with poor overall survival (hazard ratio: 3.53, 95% confidence interval: 1.35–9.24, *p* = 0.01), advanced stage disease (*p* = 0.045), deep myometrial invasion (*p* = 0.045), and distant metastasis (*p* < 0.01). In EC with deep myometrial invasion, matrix metalloproteinase (MMP) 2 was highly expressed along with LSR. Anti-LSR mAb significantly inhibited the tumor growth in EC cell xenograft mouse model (tumor volume, 407.1 mm^3^ *versus* 726.3 mm^3^, *p* = 0.019). Pathway enrichment analysis identified the mitogen-activated protein kinase (MAPK) pathway as a signaling pathway associated with LSR expression. Anti-LSR mAb suppressed the activity of MAPK in vivo. In vitro assays using EC cell lines demonstrated that LSR regulated cell proliferation, invasion, and migration through MAPK signaling, particularly MEK/ERK signaling and membrane-type 1 MMP (MT1-MMP) and MMP2. Moreover, ERK1/2-knockdown suppressed cell proliferation, invasion, migration, and the expression of MT1-MMP and MMP2.

**Conclusions:**

Our results suggest that LSR contributes to tumor growth, invasion, metastasis, and poor prognosis of EC through MAPK signaling. Anti-LSR mAb is a potential therapeutic agent for EC.

**Supplementary Information:**

The online version contains supplementary material available at 10.1186/s12885-022-09789-6.

## Background

Endometrial cancer (EC) is the fifth most common malignancy in women [1]. Patients with early-stage EC (stage I–II, according to the 2008 International Federation of Gynecology and Obstetrics [FIGO] staging system [2]) have a relatively favorable prognosis, whereas patients with advanced stage (stage III–IV) and recurrent EC have a poor prognosis [1, 3, 4]. The primary treatment for EC is surgery, including total hysterectomy as a standard procedure [5]. For patients with advanced and recurrent EC, surgical treatment is frequently challenging due to tumor enlargement, intraperitoneal dissemination, or metastasis; thus, chemotherapy remains a mainstay of EC treatment [6]. Nevertheless, the efficacy of current chemotherapy is limited. Particularly, the response rate to second-line chemotherapy for recurrent EC is approximately 25%, and few regimens have proven effective for advanced and recurrent EC [4, 7, 8]. For gynecologic malignancies, several molecular targeted agents have been used in recent years. Bevacizumab, which is an anti-vascular endothelial growth factor monoclonal antibody (mAb), has been shown to be effective against advanced ovarian cancer (OC) [9]. However, its antitumor effect on EC has not been demonstrated in phase III clinical trials. Therefore, it is necessary to identify new target molecules and develop new therapeutic agents for EC.

We previously identified lipolysis-stimulated lipoprotein receptor (LSR) as a highly expressed molecule in OC cells using the isobaric tags for relative and absolute quantitation labeling method [10]. LSR is a transmembrane protein consisting of 581 amino acids that constructs tricellular tight junctions [11, 12]. In addition, LSR plays an important role in the metabolism of triglyceride-rich lipoproteins, primarily in the liver [13, 14]. We showed that patients with high-LSR expression had a poor prognosis in OC and gastric cancer [10, 15]. Furthermore, we demonstrated that an anti-LSR mAb which we developed inhibited tumor growth involved in lipid uptake, and that its antitumor effect was a direct manner, independent of antibody-dependent cellular cytotoxicity and complement-dependent cytotoxicity [10, 15]. EC has the same histopathological subtypes as OC, such as endometrioid and serous carcinoma, and their protein expression profiles are similar across the organs [16]. Therefore, we focused on LSR as a candidate for a novel therapeutic target for EC and conducted a preclinical study using an anti-LSR mAb that we developed.

The primary aim of the present study was to examine whether an anti-LSR mAb could be a novel therapeutic agent for EC. The secondary aim was to evaluate the role of LSR on the prognosis of patients with EC and to investigate the function of LSR in EC cells.

## Methods

Detailed and additional information are provided in Supplemental method S1.

### Patients and tissue samples

Tissue samples were prepared from 228 patients with EC who underwent a hysterectomy between 2006 and 2015. Patients with relatively rare histological subtypes, such as clear cell and mucinous carcinoma, and patients who received preoperative radiation therapy or chemotherapy were excluded from this study. This study was approved by the Osaka University Research Ethics Committee (No. 19241). We obtained a written informed consent from all patients and conducted this study in accordance with the Declaration of Helsinki.

### Immunohistochemical analysis

Immunohistochemistry (IHC) was performed using a Dako REAL EnVision Detection System (K5007). Three gynecologic oncologists trained in pathological diagnosis individually evaluated the staining intensity and distribution of tumor tissue which was scored as follows: 1 (weakly stained throughout the lesion), 2 (strongly stained in < 25% of the lesion), 3 (strongly stained in 25%–50% of the lesion), and 4 (strongly stained in > 50% of the lesion). Similar to our previous reports, scores of 1–2 were classified as a low expression group and scores of 3–4 were placed in a high expression group [10, 15].

### Cell lines and cultures

Two human endometrioid carcinoma cell lines (HEC1 and HEC116) were provided by the Japanese Collection of Research Bioresources (Osaka, Japan) and were cultured in Dulbecco’s modified Eagle’s medium (Nacalai tesque, Kyoto, Japan) containing 10% fetal bovine serum (Gibco, Grand Island, NY, USA). Both cell lines were authenticated by genetic profiling using polymorphic short tandem repeat analysis.

### Small interfering RNA transfection

Cells were transfected with small interfering RNA (siRNA) using Lipofectamine RNAiMAX Transfection Reagent (Invitrogen, CA, USA) according to the manufacture’s protocol. We used LSR-siRNA (L-009672–00, Dharmacon, CO, USA), ERK1/2-siRNA (#6560, Cell Signaling Technology, MA, USA), and control-siRNA (D-001810–10, Dharmacon, CO, USA).

### Western blot analysis

We performed western blot analysis using the sodium dodecyl sulfate–polyacrylamide gel electrophoresis [17]. Primary antibodies which we used can be found on Supplemental method S1.

### Cell proliferation assay

We performed the WST-8 assay at 72, 96, and 120 h following siRNA transfection using Cell Count Reagent SF (Nacalai Tesque, Kyoto, Japan).

### Cell invasion assay

CytoSelect cell invasion assay kit (CBA-112, Cell Biolabs, CA, USA) was used according to the manufacture’s instruction. After suspending the cells at a concentration of 1.0 × 10^5^ cells/well and incubating for 24 h, the invading cells were quantitatively evaluated by fluorescence measurement using CyQuant GR Dye (Invitrogen, CA, USA).

### Cell migration assay

Cell migration assays were conducted using transwell chambers with an 8.0 μm pore (Corning, Falcon, NY, USA). After suspending the cells at a concentration of 4.0 × 10^4^ cells/well and incubating for 24 h, the migrating cells attached to the bottom of the membrane were stained with Giemsa and imaged (200 X) in three microscopic fields per well using a fluorescence microscope (BZ-X710, Keyence, Osaka, Japan).

### Pathway enrichment and ontology analysis

Pathway enrichment and ontology analysis was performed using a published protein expression data of EC samples [18]. After excluding normal endometrial samples from this dataset, the expression data of 10,999 proteins were re-standardized and analyzed in 95 EC samples. Proteins lacking expression data were excluded and 8,017 proteins were finally included in the analysis. In the high-LSR sample group, which included 24 EC samples with LSR expression levels in the upper quartile, expression data for 873 proteins (10.9%) that have a high correlation with LSR expression (Pearson’s correlation coefficient ≤  − 0.4 or ≥ 0.4) were analyzed using the Database for Annotation, Visualization, and Integrated Discovery [19, 20].

### Antibody therapy in a xenograft mouse model

All animal experiments were carried out according to the Institutional Ethical Guidelines for Animal Experimentation at Osaka University and reported in accordance with Animal Research: Reporting of In Vivo Experiments guidelines [21].

For the development of a xenograft model, 2.0 × 10^6^ HEC1 cells were injected into Institute of Cancer Research nu/nu mice subcutaneously. Tumor volumes were evaluated twice a week [length × width^2^ × 0.5]. When the mean tumor volume reached approximately 100 mm^3^, the mice were randomized into two groups (5 mice per group) and received antibody therapy: an isotype control mouse IgG2a antibody (control Ab) (Sigma Aldrich, MO, USA) or chimeric chicken–mouse anti-LSR mAb (#1–25; Pharmafoods). These antibodies were administrated intraperitoneally at a dose of 200 μg/body twice a week for 3 weeks. IHC staining in resected tumors was performed using anti-phospho-ERK1/2 antibody (Thr202/Tyr204) (#4370), anti-Ki-67 antibody (#9027), or anti-cleaved caspase-3 antibody (#9661) from Cell Signaling Technology (MA, USA). IHC-positive cells were imaged (400 X) and counted in three microscopic fields per lesion using a fluorescence microscope (BZ-X710) and BZ-X Analyzer software from Keyence (Osaka, Japan).

### Statistical analysis

The data are shown as means ± standard deviations for in vitro analysis and means ± standard errors of the means for in vivo analysis. We used Chi-square or Fisher’s exact test for categorical variables and a Student’s t-test for continuous variables. Overall survival (OS) was evaluated using the Kaplan–Meier method and compared using the log-rank test. The Cox proportional-hazards regression model was used for multivariate analysis to determine poor prognostic factors in EC. Variables included LSR expression, histological grade, tumor involvement of adnexa or serosa, lymph node metastasis, distant metastasis, and FIGO stage. All statistical analyses were conducted using JMP Pro version 15.1.0 software (SAS Institute, NC, USA). *P*-values < 0.05 were considered statistically significant.

## Results

### High expression of LSR is associated with poor prognosis in patients with EC

**Fig. 1:**
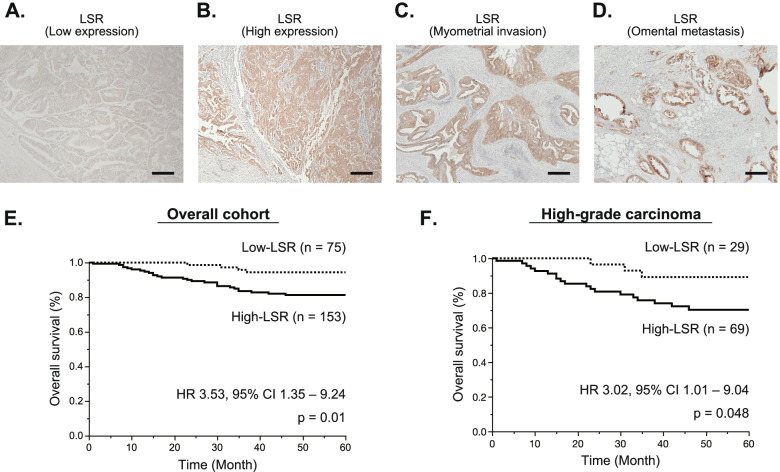
Survival analysis of patients with endometrial cancer using immunohistochemistry for lipolysis-stimulated lipoprotein receptor (LSR). **A** Representative immunohistochemical finding of low-LSR expression; score 1 (weakly stained throughout the lesion). Scale bar, 200 μm. **B** Representative immunohistochemical finding of high-LSR expression; score 4 (strongly stained in > 50% of the lesion). Scale bar, 200 μm. **C** LSR was also highly expressed in lesions with myometrial invasion. Scale bar, 200 μm. **D** In omental metastatic lesions, high expression of LSR was observed. Scale bar, 200 μm. **E** In overall cohort (all histological subtypes), the 5-year overall survival rate in the high-LSR expression group (*n* = 153) was significantly lower compared with that in the low-LSR expression group (*n* = 75) (hazard ratio [HR]: 3.53, 95% confidence interval [CI]: 1.35–9.24, *p* = 0.01). **F** In high-grade carcinoma (grade 3 endometrioid and serous carcinoma), patients in the high-LSR expression group (*n* = 69) had a significantly poorer overall survival than those in the low-LSR expression group (*n* = 29) (HR: 3.02, 95% CI: 1.01–9.04, *p* = 0.048)

Representative IHC findings of low- and high-LSR expression are shown in Fig. 1A (score 1) and 1B (score 4). For patients with EC showing high-LSR expression, LSR was also highly expressed in lesions with myometrial invasion (Fig. 1C) and omental metastatic lesions (Fig. 1D).

Patients were classified into two groups based on IHC score: a high-LSR expression group (*n* = 153) and a low-LSR expression group (*n* = 75). The median follow-up period was 62 months (interquartile range: 42.5–78 months), including 35 deaths. The patient characteristics and clinicopathological features are shown in Table 1. In the high-LSR expression group, there were significantly more patients with advanced stage disease (stage III or IV; 37.3% *versus* 24.0%, *p* = 0.045), tumor involvement of the adnexa or serosa (26.8% *versus* 6.7%, *p* < 0.01), and distant metastasis (13.7% *versus* 1.3%, *p* < 0.01) compared with the low-LSR expression group. Moreover, in the high-LSR expression group, the patients with invasion of more than 1/2 of the myometrium tended to be evaluated more frequently (47.7% *versus* 38.7%, *p* = 0.20), in particular, the patients with deep invasion of more than 3/4 of the myometrium were observed significantly more frequently (37.3% *versus* 24.0%, *p* = 0.045) compared with the low-LSR expression group. The rate of high-LSR expression did not vary significantly between low-grade carcinoma (grade 1 and 2 endometrioid carcinoma) and high-grade carcinoma (grade 3 endometrioid and serous carcinoma) (64.6% [84/130] *versus* 70.4% [69/98], *p* = 0.39), suggesting that LSR is highly expressed in EC regardless of the tumor grade.

**Table 1:** Characteristics and clinicopathological features of patients with endometrial cancer per LSR expression

Characteristics	Low-LSR	High-LSR	*p* value
Number of cases	75	153	
Age, years	57 (52–66)	62 (53–69)	0.15
BMI	21.8 (19.6–25.1)	22.0 (19.6–25.1)	0.77
Histological subtype			0.061
Endometrioid, Grade 1	26 (34.7)	40 (26.1)	
Endometrioid, Grade 2	20 (26.7)	44 (28.8)	
Endometrioid, Grade 3	22 (29.3)	34 (22.2)	
Serous	7 (9.3)	35 (22.9)	
FIGO stage			0.045
I–II	57 (76.0)	96 (62.7)	
III–IV	18 (24.0)	57 (37.3)	
Depth of myometrial invasion			
≥ 50% of myometrium	29 (38.7)	73 (47.7)	0.20
≥ 75% of myometrium	18 (24.0)	57 (37.3)	0.045
Tumor involvement of adnexa or serosa	5 (6.7)	41 (26.8)	0.0002
LVSI	32 (42.7)	74 (48.4)	0.48
Lymph node metastasis	14 (18.7)	37 (24.2)	0.40
Distant metastasis	1 (1.3)	21 (13.7)	0.0016

Number (% per group) or median (IQR; interquartile range) is shown

*Abbreviations: LSR* Lipolysis-stimulated lipoprotein receptor, *BMI* body mass index, *FIGO* the International Federation of Gynecology and Obstetrics, and *LVSI* lymphovascular space invasion

Survival analysis indicated that the 5-year OS rate in the high-LSR expression group was significantly lower compared with that in the low-LSR expression group (hazard ratio [HR]: 3.53, 95% confidence interval [CI]: 1.35–9.24, *p* = 0.01; Fig. 1E). In low-grade carcinoma, there was no significant difference in OS rate between patients with high-LSR expression and those with low-LSR expression (HR: 5.09, 95% CI: 0.64 − 40.2, *p* = 0.12). On the other hand, in high-grade carcinoma, patients in the high-LSR expression group had a significantly poorer prognosis than those in the low-LSR expression group (HR: 3.02, 95% CI: 1.01–9.04, *p* = 0.048; Fig. 1F). These results suggest that although LSR expression is not related to the tumor grade, the effect of high expression of LSR is greater in high-grade carcinoma than in low-grade carcinoma.

The multivariate analysis identified distant metastasis (adjusted HR: 4.49, 95% CI: 1.93 − 10.5, *p* < 0.01) and FIGO stage (adjusted HR: 3.84, 95% CI: 1.11 − 13.3, *p* = 0.034) as independent prognostic factors in EC (Supplemental Table S1). Thus, we verified the significance of LSR in subgroups focused on tumor invasion and metastasis, which define the FIGO stage. In a subgroup of patients with myometrial invasion more than 50%, patients in the high-LSR expression group (*n* = 73) had a significantly poorer OS rate than those in the low-LSR expression group (*n* = 29) (HR: 3.58, 95% CI: 1.07–11.98, *p* = 0.038; Supplemental Fig. S1A). Additionally, in a subgroup of patients with myometrial invasion more than 50% or distant metastasis, the OS rate in the high-LSR expression group (*n* = 75) was significantly lower compared with that in the low-LSR expression group (*n* = 29) (HR 3.67, 95%CI: 1.1–12.2, *p* = 0.034; Supplemental Fig. S1B). Therefore, we believe that although LSR is not an independent poor prognostic factor, it strongly influences the FIGO stage defined by tumor invasion and/or metastasis and contributes to a poor prognosis in EC.

### Anti-LSR monoclonal antibody shows a significant antitumor effect against EC in a xenograft model

**Fig. 2:**
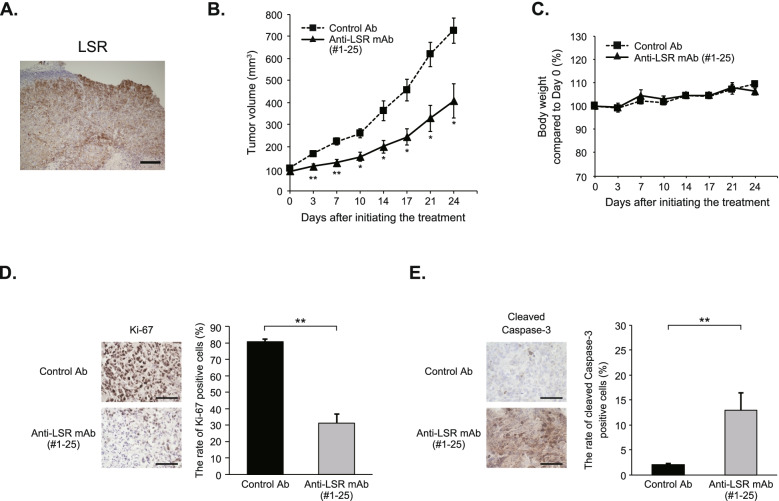
Anti-LSR monoclonal antibody shows a significant antitumor effect against endometrial cancer in a xenograft model. Healthy female 6-week-old nude mice were subcutaneously inoculated with 2.0 X 10^6^ HEC1 cells. When the tumor volume reached approximately 100 mm^3^, the mice were randomized into two groups (5 mice per group) and administrated with isotype control mouse IgG2a antibody (control Ab) or anti-LSR monoclonal antibody (anti-LSR mAb, #1–25) twice a week for 3 weeks. **A** Expression of LSR in HEC1-xenograft tumors in nude mice was confirmed by immunohistochemistry. Scale bar, 200 μm. **B** Anti-LSR mAb significantly inhibited tumor growth compared with the control Ab (mean tumor volume, 407.1 mm^3^ *versus* 726.3 mm^3^, *p* = 0.019). Data were shown as means ± standard errors of the means (*n* = 5; *p < 0.05, **p < 0.01). **C** No significant weight loss was observed in the mice following anti-LSR mAb administration. **D** The rate of Ki-67-positive cells was significantly decreased in the anti-LSR mAb-treated group compared with the control Ab-treated group (31.1% *versus* 80.8%, *p* < 0.01). Magnification, 400 X; Scale bar, 100 μm. Data were shown as means ± standard deviations (*n* = 5; **p < 0.01). **E** The rate of cleaved caspase-3-positive cells was significantly increased in the anti-LSR mAb-treated group compared with that in the control Ab-treated group (12.9% *versus* 2.0%, *p* < 0.01). These results suggest that our anti-LSR mAb exerts a significant antitumor effect associated with apoptosis in vivo. Magnification, 400 X; Scale bar, 100 μm. Data were shown as means ± standard deviations (*n* = 5; **p < 0.01)

We developed a chimeric chicken–mouse anti-LSR mAb (#1–25) and conducted a preclinical study on its efficacy. We confirmed the expression of LSR in HEC1-xenograft tumors in nude mice by IHC (Fig. 2A). Mice were injected intraperitoneally with control Ab or anti-LSR mAb at a dose of 200 μg/body twice a week for 3 weeks. Anti-LSR mAb significantly inhibited tumor growth compared with control Ab (mean tumor volume, 407.1 mm^3^ *versus* 726.3 mm^3^, *p* = 0.019; Fig. 2B). No significant weight loss was observed in the mice following anti-LSR mAb administration (Fig. 2C).

IHC for Ki-67, which has been reported as a cell proliferation marker [22], demonstrated that the rate of Ki-67-positive cells was significantly decreased in the anti-LSR mAb-treated group compared with the control Ab-treated group (31.1% *versus* 80.8%, *p* < 0.01; Fig. 2D). We evaluated the expression of cleaved caspase-3 as an apoptosis-related protein in xenograft tumors using IHC [23]. The rate of cleaved caspase-3-positive cells was significantly increased in the anti-LSR mAb-treated group compared with that in the control Ab-treated group (12.9% *versus* 2.0%, *p* < 0.01; Fig. 2E). These results suggest that our anti-LSR mAb exerts a significant antitumor effect associated with apoptosis in vivo, which indicates its potential to be a novel therapeutic agent for EC.

### High expression of LSR is associated with the MAPK signaling pathway in a bioinformatic analysis

While our preclinical experiments showed the antitumor activity of anti-LSR mAb, the molecular function of LSR in EC has been largely unknown. Therefore, we performed a pathway enrichment and ontology analysis using protein expression data of EC samples [18]. Pathway analysis revealed that proteins correlating with high-LSR expression were enriched in 23 pathways (Supplemental Table S2). Of these 23 pathways, the mitogen-activated protein kinase (MAPK) signaling pathway was the only pathway involved in cell proliferation [24–26]. In addition, an ontology analysis revealed that proteins associated with high-LSR expression were enriched in “regulation of ERK1 and ERK2 cascade (*p* < 0.01)” and “regulation of MAPK cascade (*p* = 0.025)” (Supplemental Table S3).

### LSR regulates cell proliferation and tumor growth through the MEK/ERK signaling pathway

**Fig. 3:**
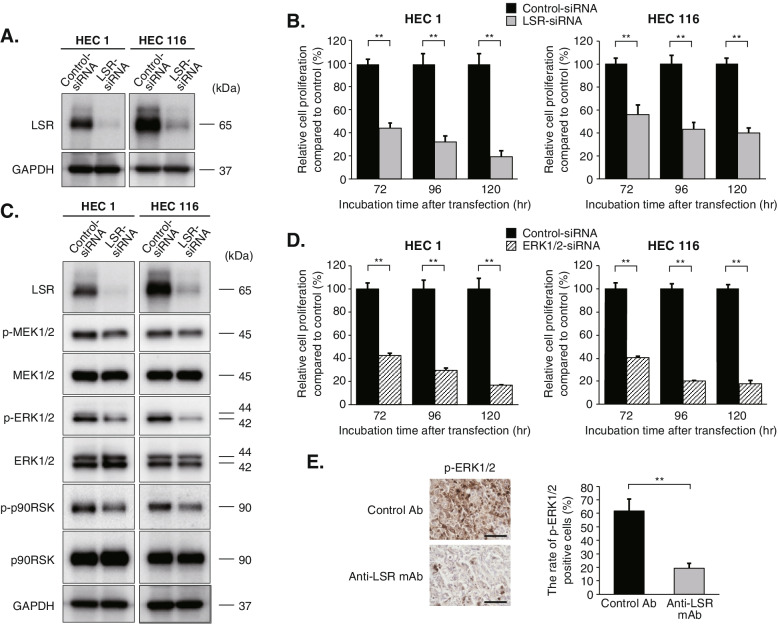
LSR regulates cell proliferation and tumor growth through the MEK/ERK signaling pathway in endometrial cancer. **A** LSR-knockdown cells were generated by transfecting endometrial cancer cell lines (HEC1 and HEC116) with LSR-siRNA. We confirmed that the expression of LSR was suppressed in LSR-knockdown cells using western blot analysis. (The images of the western blot bands were cropped from the images shown in Supplemental Fig. S3A and B). **B** Cell proliferation was evaluated using the WST-8 assay at 72, 96, and 120 h following transfection. Cell proliferation was significantly suppressed in LSR-knockdown cells. Data were shown as means ± standard deviations (**p < 0.01). **C** Western blot analysis showed that in the MEK/ERK signaling pathway, the phosphorylation of MEK1/2, ERK1/2, and p90RSK was suppressed in LSR-knockdown cells. (The images of the western blot bands were cropped from the images shown in Supplemental Fig. S3C and D). **D** ERK1/2-knockdown also suppressed cell proliferation in HEC1 and HEC116 cells. Data were shown as means ± standard deviations (**p < 0.01). **E** Immunohistochemical analysis for phospho-ERK1/2 was performed using HEC1-xenograft tumors in nude mice. The rate of phospho-ERK1/2-positive cells was significantly lower in the anti-LSR mAb-treated group than in the control Ab-treated group (19.3% *versus* 61.8%, *p* < 0.01). Magnification, 400 X; Scale bar, 100 μm. Data were shown as means ± standard deviations (*n* = 5; **p < 0.01). Abbreviation: anti-LSR mAb, anti-LSR monoclonal antibody; control Ab, isotype control mouse IgG2a antibody; hr, hour; p-ERK1/2, phospho-ERK1/2; p-MEK1/2, phospho-MEK1/2; p-p90RSK, phospho-p90RSK

Based on the results of pathway enrichment and ontology analysis, we investigated the molecular function of LSR, focusing on the MAPK pathway, especially the MEK/ERK pathway. LSR-knockdown cells were generated by transfecting EC cell lines with LSR-siRNA (Fig. 3A). The WST-8 assay showed that cell proliferation was significantly suppressed in LSR-knockdown cells (*p* < 0.01, respectively; Fig. 3B). In the MEK/ERK signaling pathway, the phosphorylation of MEK1/2, ERK1/2, and p90RSK was suppressed in LSR-knockdown cells (Fig. 3C). No obvious changes of protein expression resulting from LSR-knockdown were observed in other MAPK pathways (SAPK/JNK and p38 MAPK pathways) (Supplemental Fig. S2). In addition, ERK1/2-knockdown significantly suppressed cell proliferation in HEC1 and HEC116 cells (*p* < 0.01, respectively; Fig. 3D). IHC analysis using HEC1-xenograft tumors in mice showed that the rate of phospho-ERK1/2-positive cells was significantly lower in the anti-LSR mAb-treated group than in the control Ab-treated group (19.3% *versus* 61.8%, *p* < 0.01; Fig. 3E). These results indicate that LSR regulates cell proliferation and tumor growth through the MEK/ERK signaling pathway in EC.

### LSR regulates cell invasion and migration through MEK/ERK signaling and subsequent matrix metalloproteinases

**Fig. 4:**
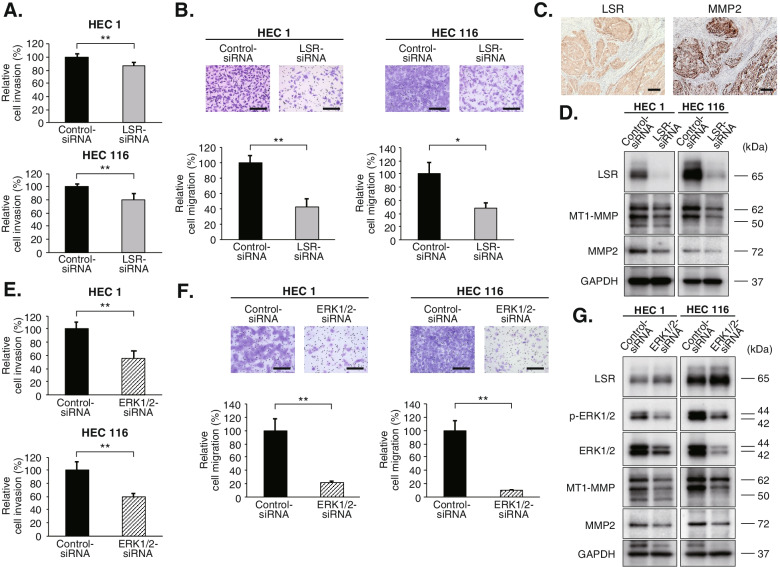
LSR regulates cell invasion and migration through MEK/ERK signaling and subsequent matrix metalloproteinases. **A** Cell invasion was downregulated by LSR-knockdown in HEC1 and HEC116 cells. Data were shown as means ± standard deviations (**p < 0.01). **B** LSR-knockdown suppressed cell migration in HEC1 and HEC116 cells. Magnification, 200 X; Scale bar, 200 μm. Data were shown as means ± standard deviations (*n* = 3; *p < 0.05, **p < 0.01). **C** Immunohistochemical analysis revealed that MMP2 was highly expressed along with LSR in endometrial cancer lesions with deep myometrial invasion. Scale bar, 200 μm. **D** Western blot analysis showed that the expression of MT1-MMP and MMP2 was suppressed in LSR-knockdown cells. (The images of the western blot bands were cropped from the images shown in Supplemental Fig. S3E and F). **E** Cell invasion was downregulated by ERK1/2-knockdown in both HEC1 and HEC116 cells. Data were shown as means ± standard deviations (**p < 0.01). **F** ERK1/2-knockdown suppressed cell migration in HEC1 and HEC116 cells. Magnification, 200 X; Scale bar, 200 μm. Data were shown as means ± standard deviations (*n* = 3; **p < 0.01). **G** Western blot analysis showed that the expression of MT1-MMP and MMP2 was suppressed by ERK1/2-knockdown in HEC1 and HEC116. These results indicate that LSR regulates cell proliferation, invasion, and migration through the MEK/ERK signaling pathway and subsequent MT1-MMP and MMP2 in endometrial cancer. (The images of the western blot bands were cropped from the images shown in Supplemental Fig. S3G and H). Abbreviation: MMP, matrix metalloproteinase; p-ERK1/2, phospho-ERK1/2

Since IHC analysis using clinical samples revealed that high-LSR expression was significantly associated with myometrial invasion and metastasis (Table 1), we investigated the role of LSR on EC cell invasion and migration. The invasion and migration assays showed that LSR-knockdown downregulated cell invasion and migration in HEC1 and HEC116 cells (*p* < 0.05, respectively; Fig. 4A and B).

**Fig. 5:**
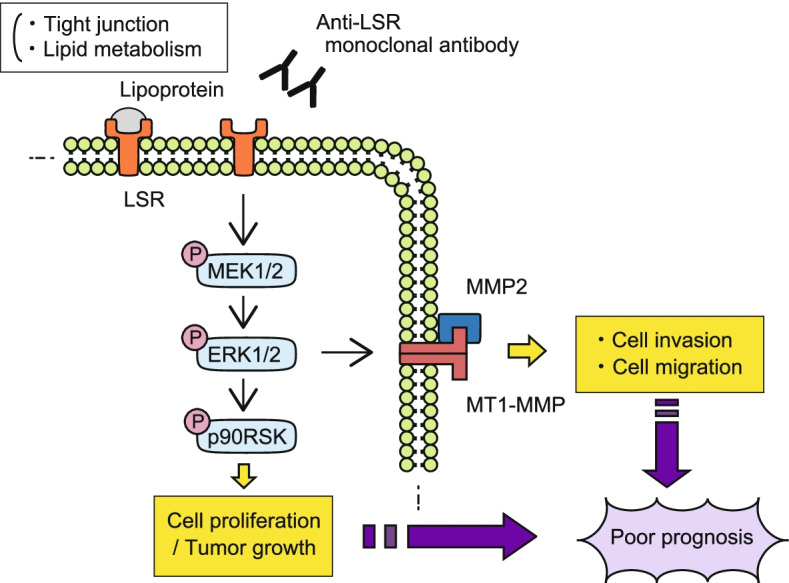
A schema of the function and role of LSR in endometrial cancer. LSR is highly expressed in endometrial cancer cells and regulates cell proliferation, invasion, and migration through the MEK/ERK signaling pathway and subsequent MT1-MMP and MMP2. By these mechanisms, LSR contributes to a poor prognosis of patients with endometrial cancer. The newly developed anti-LSR monoclonal antibody which exerted a significant antitumor effect in the xenograft mouse model has the potential to be a novel therapeutic agent for endometrial cancer

Next, to clarify the mechanism through which LSR contributes to the invasion and migration of EC cells, we investigated the relationship between LSR and matrix metalloproteinases (MMPs). IHC analysis revealed that MMP2 was highly expressed along with LSR in EC lesions with deep myometrial invasion (Fig. 4C). Western blot analysis showed that the expression of membrane-type 1 MMP (MT1-MMP) and MMP2 was suppressed in LSR-knockdown cells (Fig. 4D). Furthermore, we investigated the relationship between the MEK/ERK signaling pathway and cell invasion, migration, and the expression of MMPs in EC cells. The invasion and migration assays demonstrated that ERK1/2-knockdown suppressed cell invasion and migration in HEC1 and HEC116 cells (*p* < 0.01; Fig. 4E and F). ERK1/2-knockdown also suppressed the expression of MT1-MMP and MMP2 as determined by western blot analysis (Fig. 4G). These results indicate that LSR regulates cell proliferation, invasion, and migration through the MEK/ERK signaling pathway and subsequent MT1-MMP and MMP2 in EC (Fig. 5).

## Discussion

Key findings of our study were that LSR was associated with tumor growth, invasion, metastasis, and poor prognosis through MAPK signaling in EC, and that our anti-LSR mAb had a potential to be a novel therapeutic agent for EC (Fig. 5).

The function and role of LSR has been studied in various cancers including that of the breast, bladder, and colon [10, 15, 27–29]. Although there have been some reports regarding LSR in EC [30, 31], to the best of our knowledge, studies using multiple cell lines or survival analysis using clinical samples have not been conducted. Our in vitro/in vivo assays and pathway enrichment analysis showed that LSR contributed to aggressive behaviors of EC cells, such as cell proliferation, invasion, and migration, via the MAPK signaling pathway. Furthermore, we showed that LSR and subsequent MAPK regulated the expression of MMPs, which are endopeptidases involved in cell invasion and migration of various cancers by degrading the basement membrane and extracellular matrix [32–34]. Several studies in prostate cancer, sarcoma, and fibroblast cells have shown that activation of MEK/ERK signaling promoted the expression of MT1-MMP and MMP2 [35–38]. In this study, we demonstrated a novel pathway originating from a membrane protein that promotes MAPK-mediated cell proliferation, invasion, and migration in EC.

Our anti-LSR mAb inhibited MAPK signaling and showed a significant antitumor effect associated with apoptosis in the EC cell xenograft mouse model. As reported previously, our anti-LSR mAb did not show any side effects in blood parameters such as complete blood cell count, serum chemistry, or lipid metabolites [10]. Additionally, no pathological abnormality was noted in normal mouse organs [10]. MAPK inhibitors, including trametinib (MEK1/2 inhibitor) and sorafenib (multiple kinase inhibitor), have been studied and used in various malignancies [39–42]. These kinase inhibitors have not shown any significant therapeutic effects and safety, although the efficacy of these medications has been studied in EC [43, 44]. This is possibly because of the low selectivity of these kinase inhibitors for tissues and cells and MAPK gene alterations in EC (e.g., Ras/Raf mutation). Our anti-LSR mAb selectively acts on cancer cells expressing LSR and has little toxicity to normal tissues, indicating that it might be a more effective and safer agent than these kinase inhibitors.

We found that cancer cells strongly express LSR and MMP2 in invasive lesions deep in the myometrium (Fig. 4C). We speculate that this involves the tumor microenvironment, including hypoxia and glucose deprivation, which are characteristic of growing and invading solid tumors [45]. Our group has previously reported that LSR contributes to the survival of OC cells under hypoxic and hypoglycemic conditions [46]. We believe that LSR upregulates MAPK signaling and promotes cell invasion and migration via MT1-MMP and MMP2 in EC cells in this tumor microenvironment. Further evaluation of the function of LSR necessitates studies focusing on the tumor microenvironment.

Obesity is a significant risk factor for the incidence and mortality of EC [47–49]. Originally, LSR was identified as a membrane protein involved in the uptake and clearance of triglyceride-rich lipoproteins in the liver [13, 14]; however, to the best of our knowledge, its association with human obesity and hyperlipidemia has not been demonstrated yet. On the other hand, a study using the mice model reported that mice with an inactivated single LSR allele (LSR^−/+^) showed increased plasma triglyceride and cholesterol levels and gained weight [14]. Therefore, LSR might also be associated with obesity and hyperlipidemia in humans. However, we found no significant association between LSR expression levels and body mass index (BMI) in patients with EC (21.8 *versus* 22.0, *p* = 0.77; Table1). The systemic lipid metabolism or BMI of the patients might not have been affected as our study evaluated LSR expression levels of tumor tissues rather than that of systemic tissues, including the liver tissues.

There are few reports on lipid metabolism via LSR in cancer cells. We reported that LSR-positive OC cells stored more and larger lipid droplets than LSR-negative OC cells [10]. In addition, the administration of very-low-density lipoprotein (VLDL) promoted cell proliferation in LSR-positive OC cells [10]. The anti-LSR mAb inhibited tumor growth in vivo without causing hyperlipidemia and impeded cell proliferation promoted by VLDL in vitro [10]. Therefore, we believe that LSR in cancer cells has little effect on systemic changes such as obesity and hyperlipidemia, but it is involved in cellular lipid metabolism. However, several points regarding lipid metabolism via LSR in cancer cells remain unclear, and further studies are thus needed.

The strengths of this study are the following points. First, to the best of our knowledge, this study is the first to demonstrate that LSR contributes to tumor growth, invasion, metastasis, and poor prognosis through MAPK signaling in EC; this finding was obtained using a large number of clinical samples, multiple cell lines, and proteomic data. Second, we developed an anti-LSR mAb that exerted a significant antitumor effect against EC in the xenograft mouse model. Our results suggest that an anti-LSR mAb can be a novel therapeutic agent for EC. However, this is a preclinical study using a chimeric chicken–mouse antibody. Therefore, it might not reflect the efficacy and safety of anti-LSR mAb in clinical use for humans and this is the limitation of our study. Further studies using humanized anti-LSR mAb are warranted in the future.

## Conclusions

LSR is associated with tumor growth, invasion, metastasis, and poor prognosis through MAPK signaling in EC. The newly developed anti-LSR mAb has the potential to be a novel therapeutic agent for EC.

## Supplementary Information

**Additional file 1.**

**Additional file 2.**

**Additional file 3.**

**Additional file 4.**

**Additional file 5.**

**Additional file 6.**

**Additional file 7.**

## Data Availability

The data that support the findings of this study are available from the corresponding author upon reasonable request. The data that we used in the pathway enrichment and ontology analysis in this study are openly available in Supplemental Table S1 and S2 at http://doi.org/10.1016/j.cell.2020.01.026, reference number 18.

## Abbreviations

BMI: Body mass index
EC: Endometrial cancer
FIGO: International Federation of Gynecology and Obstetrics
IHC: Immunohistochemistry
LSR: Lipolysis-stimulated lipoprotein receptor
mAb: Monoclonal antibody
MAPK: Mitogen-activated protein kinase
MMP: Matrix metalloproteinase
OC: Ovarian cancer
OS: Overall survival
siRNA: Small interfering RNA
VLDL: Very-low-density lipoprotein
