# Supplementary material for: Anti-lipolysis-stimulated lipoprotein receptor monoclonal antibody as a novel therapeutic agent for endometrial cancer
Source: BMC Cancer. 2022 Jun 21;22:679. doi: 10.1186/s12885-022-09789-6 (PMC9210735; doi:10.1186/s12885-022-09789-6)

Supplemental Figure S1. Survival analysis of patients with endometrial cancer in subgroups.

A, In a subgroup of patients with myometrial invasion more than 50%, patients in the high-LSR expression group (n = 73) had a significantly poorer overall survival rate than those in the low-LSR expression group (n = 29) (hazard ratio [HR]: 3.58, 95% confidence interval [CI]: 1.07-11.98, p = 0.038).

B, In a subgroup of patients with myometrial invasion more than 50% or distant metastasis, the overall survival rate in the high-LSR expression group (n = 75) was significantly lower compared with that in the low-LSR expression group (n = 29) (HR: 3.67, 95% CI: 1.1-12.2, p=0.034)

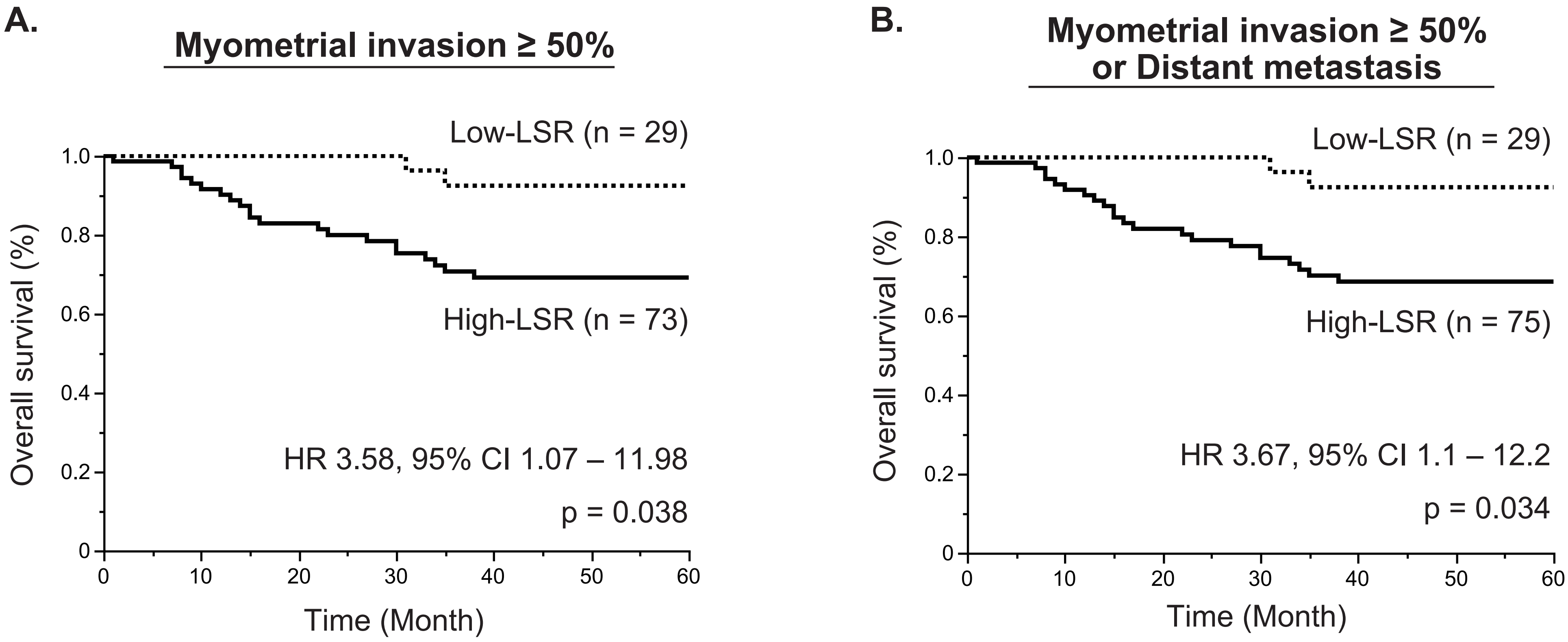

Supplement: Supplementary file 1 — Additional file 1. [file 12885_2022_9789_MOESM1_ESM.pdf]
