# Supplementary material for: Anti-lipolysis-stimulated lipoprotein receptor monoclonal antibody as a novel therapeutic agent for endometrial cancer
Source: BMC Cancer. 2022 Jun 21;22:679. doi: 10.1186/s12885-022-09789-6 (PMC9210735; doi:10.1186/s12885-022-09789-6)

Supplemental Figure S2. Western blot analysis in the MAPK signaling pathways other than the MEK/ERK pathway.

The images of the western blot bands were cropped from the images shown in Supplemental Figure S3I and S3J.

Abbreviation: p-SAPK/JNK, phospho-SAPK/JNK; p-p38 MAPK, phospho-p38 MAPK.

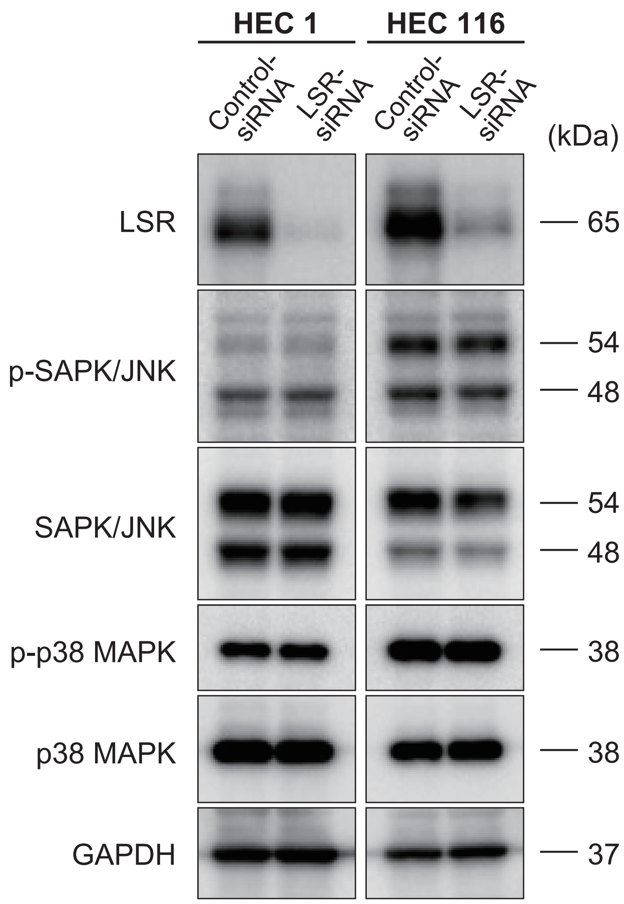

Supplement: Supplementary file 2 — Additional file 2. [file 12885_2022_9789_MOESM2_ESM.pdf]
