# Supplementary material for: Anti-lipolysis-stimulated lipoprotein receptor monoclonal antibody as a novel therapeutic agent for endometrial cancer
Source: BMC Cancer. 2022 Jun 21;22:679. doi: 10.1186/s12885-022-09789-6 (PMC9210735; doi:10.1186/s12885-022-09789-6)

Supplemental Figure S3. Original data of western blot bands used in Figure 3, Figure 4, and Supplemental Figure S2.

A. Western blot bands of LSR (i) and GAPDH (ii) after transfection of control-siRNA or LSR-siRNA in HEC1 cells (Original data of Figure 3A).

Red boxes denote the regions of original blots used in Figure 3A. Arrows indicate the edge of the membrane. Abbreviations: C, Control-siRNA; L, LSR-siRNA.

(i) LSR (65 kDa)

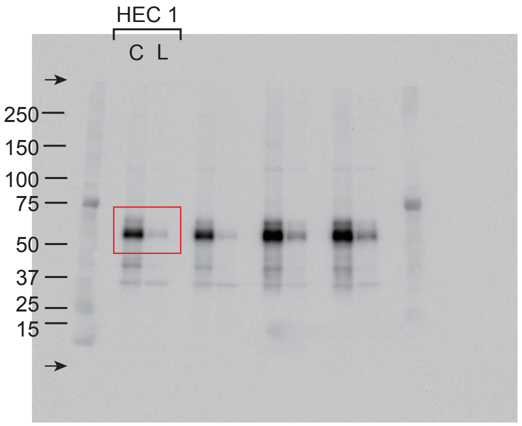

(ii) GAPDH (37 kDa)

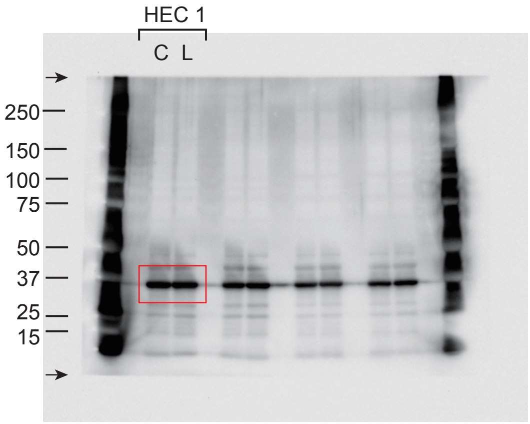

B. Western blot bands of LSR (i) and GAPDH (ii) after transfection of control-siRNA or LSR-siRNA in HEC116 cells (Original data of Figure 3A).

Red boxes denote the regions of original blots used in Figure 3A. Arrows indicate the edge of the membrane. Abbreviations: C, Control-siRNA; L, LSR-siRNA.

(i) LSR (65 kDa)

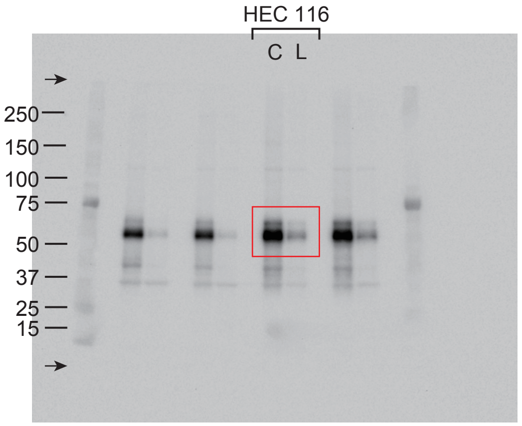

(ii) GAPDH (37 kDa)

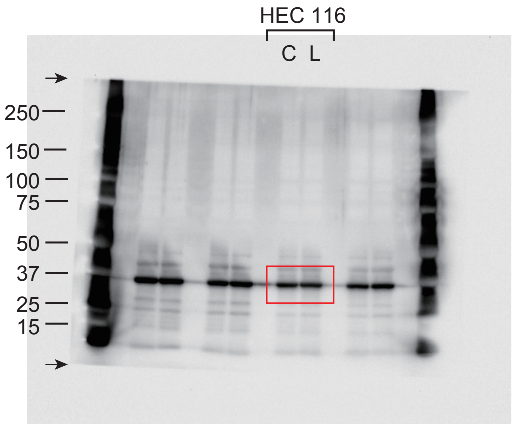

C. Western blot bands of LSR (i), phospho-MEK1/2 (ii), MEK1/2 (iii), phospho-ERK1/2 (iv), ERK1/2 (v), phospho-p90RSK (vi), p90RSK (vii), and GAPDH (viii) after transfection of control-siRNA or LSR-siRNA in HEC1 cells (Original data of Figure 3C).

Red boxes denote the regions of original blots used in Figure 3C. Arrows indicate the edge of the membrane. Abbreviations: C, Control-siRNA; L, LSR-siRNA.

(i) LSR (65 kDa)

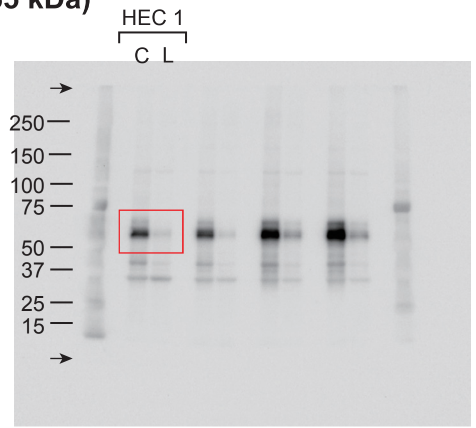

(ii) phospho-MEK1/2 (45 kDa)

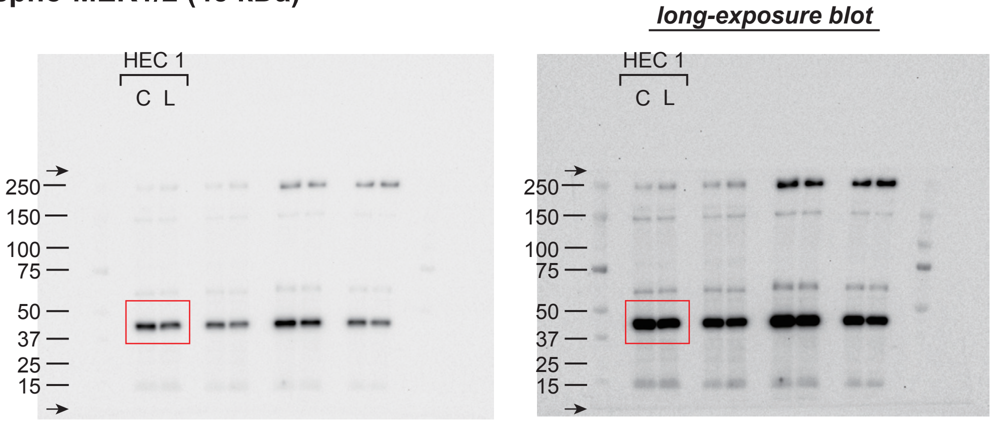

(iii) MEK1/2 (45 kDa)

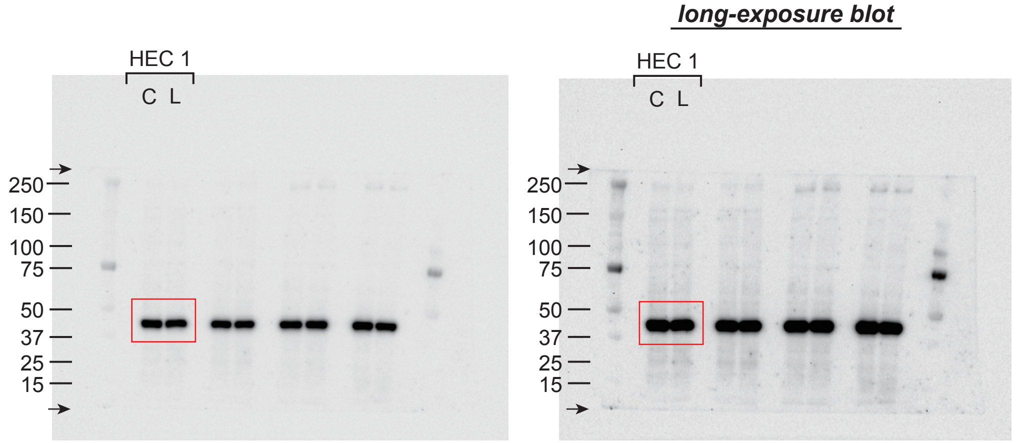

(iv) phospho-ERK1/2 (42,44 kDa)

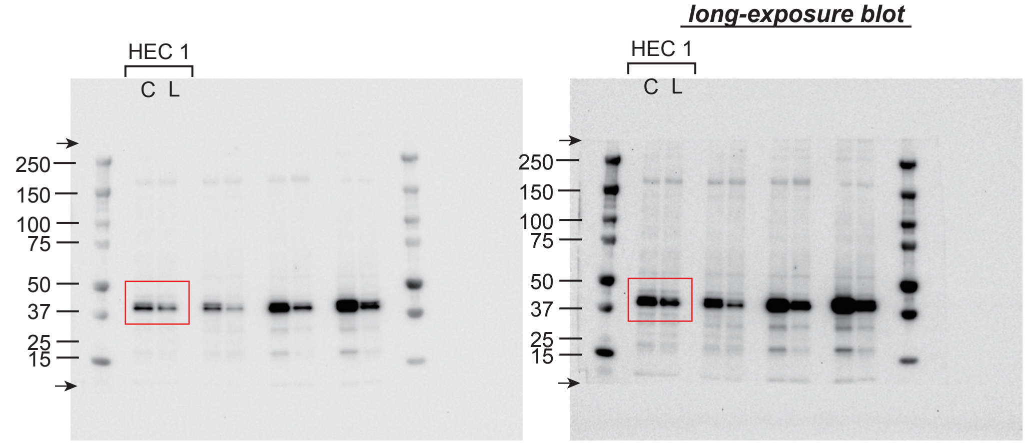

(v) ERK1/2 (42,44 kDa)

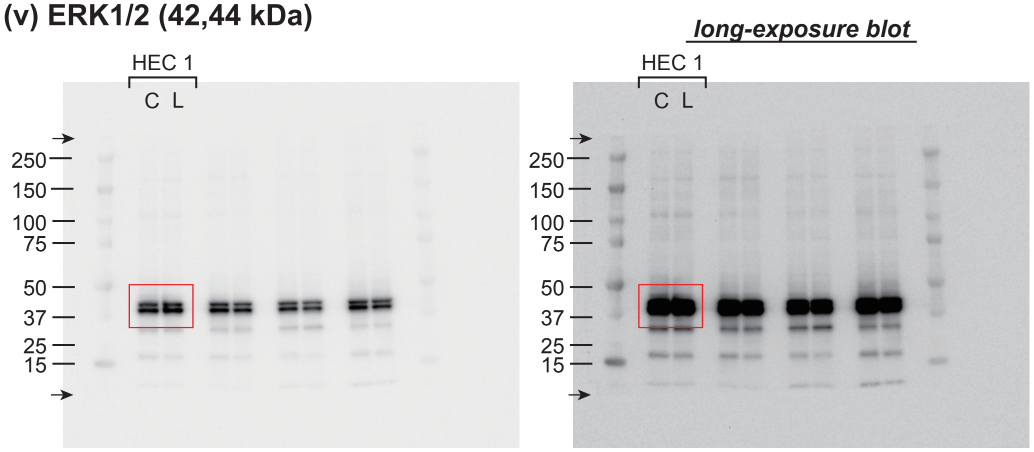

(vi) phospho-p90RSK (90 kDa)

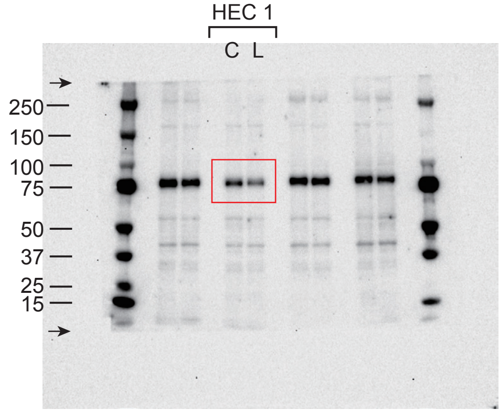

(vii) p90RSK (90 kDa)

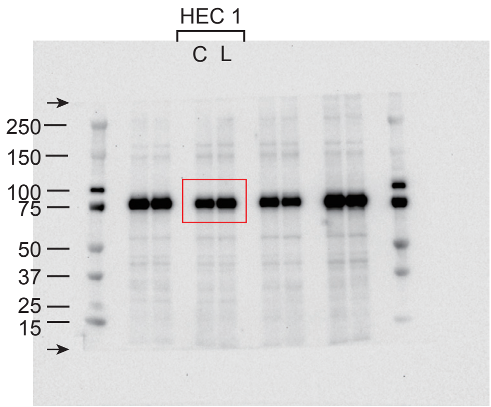

(viii) GAPDH (37 kDa)

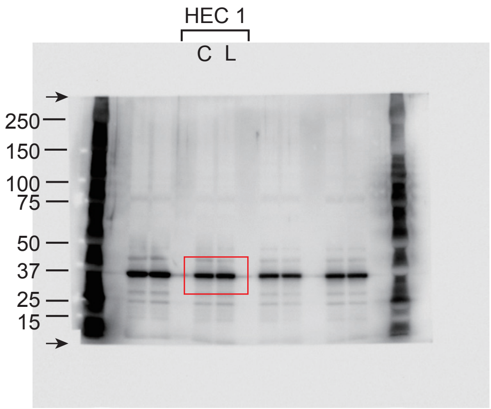

D. Western blot bands of LSR (i), phospho-MEK1/2 (ii), MEK1/2 (iii), phospho-ERK1/2 (iv), ERK1/2 (v), phospho-p90RSK (vi), p90RSK (vii), and GAPDH (viii) after transfection of control-siRNA or LSR-siRNA in HEC116 cells (Original data of Figure 3C).

Red boxes denote the regions of original blots used in Figure 3C. Arrows indicate the edge of the membrane. Abbreviations: C, Control-siRNA; L, LSR-siRNA.

(i) LSR (65 kDa)

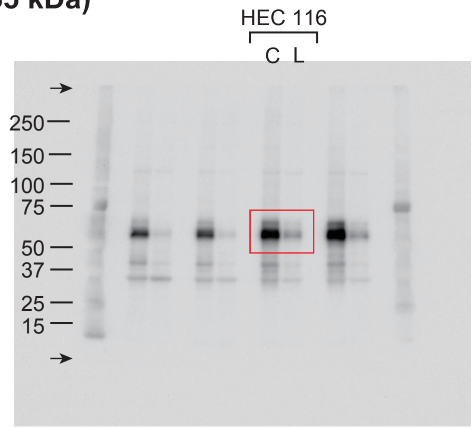

(ii) phospho-MEK1/2 (45 kDa)

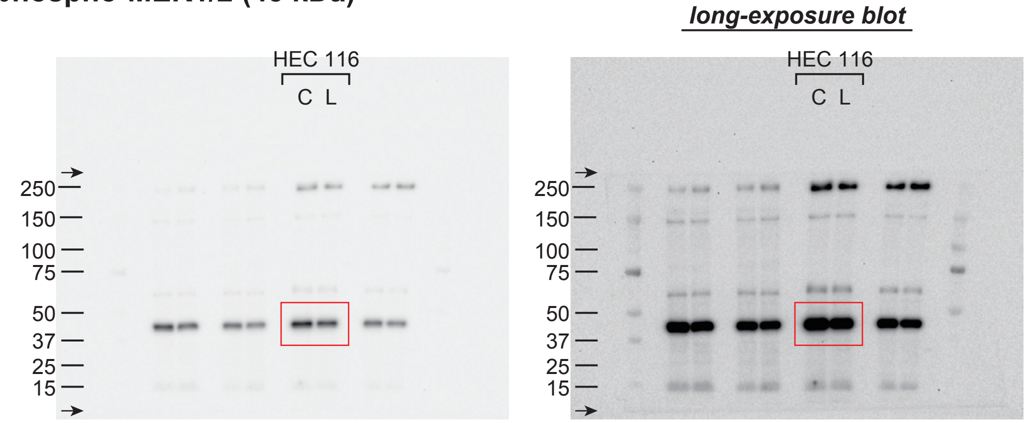

(iii) MEK1/2 (45 kDa)

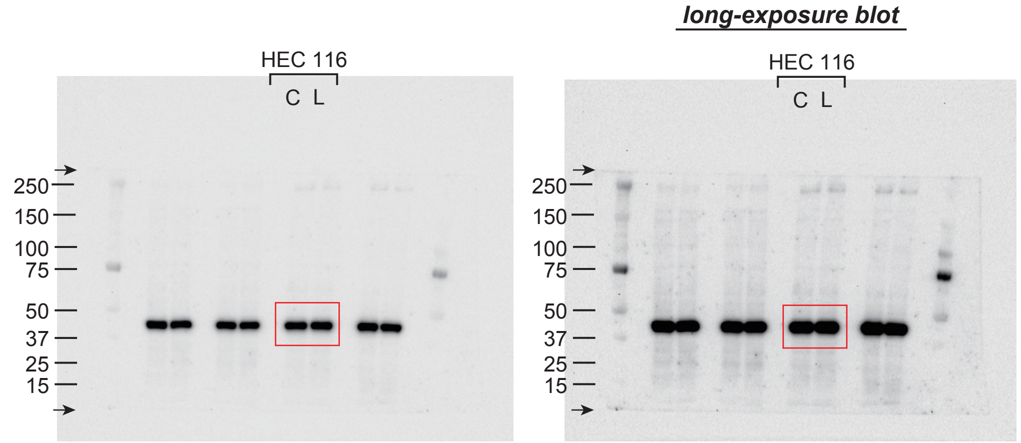

(iv) phospho-ERK1/2 (42,44 kDa)

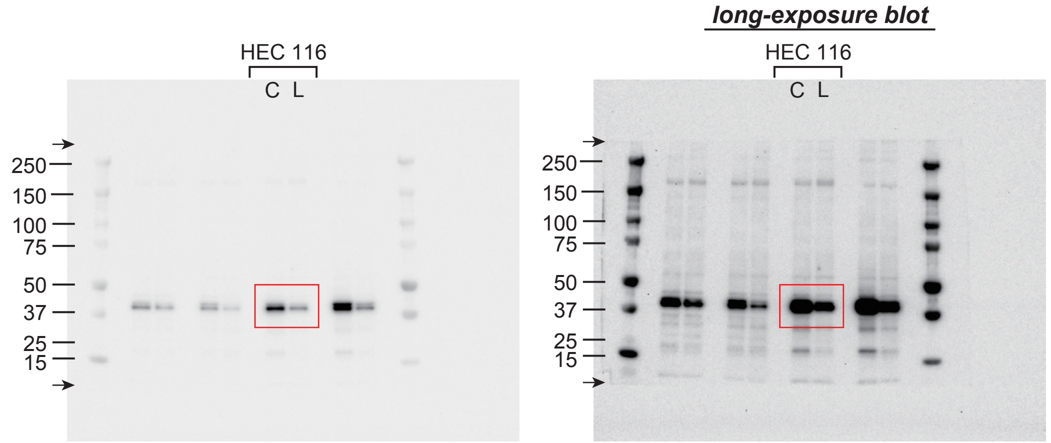

(v) ERK1/2 (42,44 kDa)

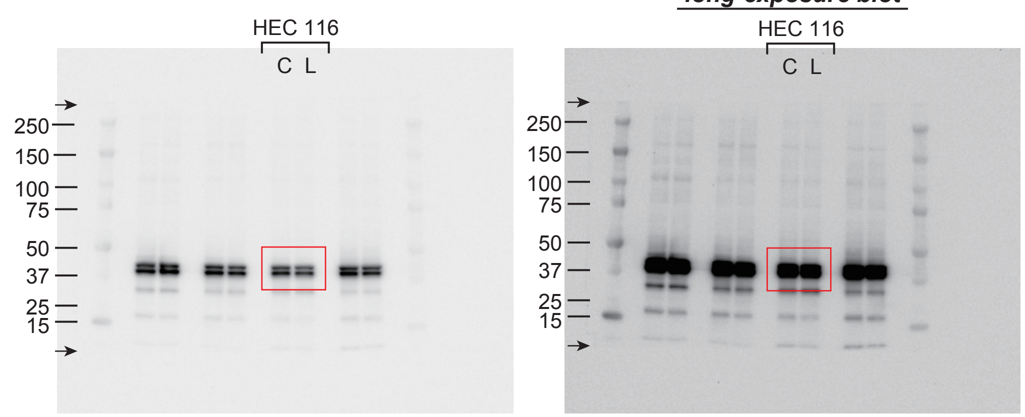

(vi) phospho-p90RSK (90 kDa)

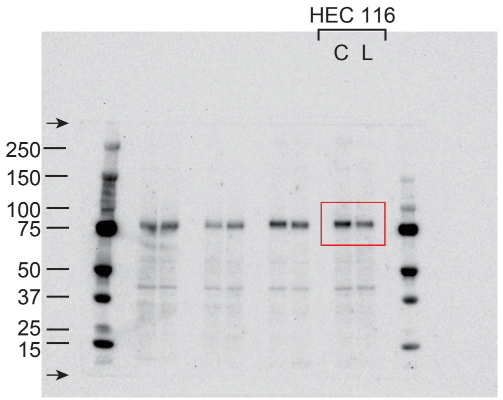

(vii) p90RSK (90 kDa)

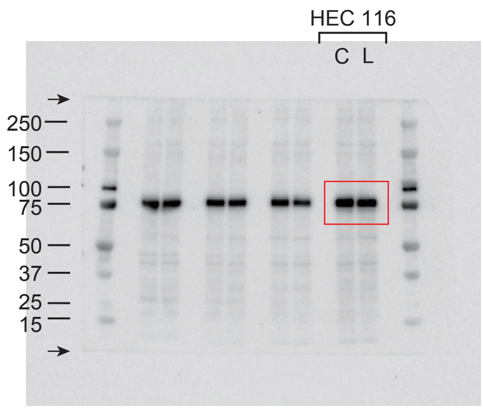

(viii) GAPDH (37 kDa)

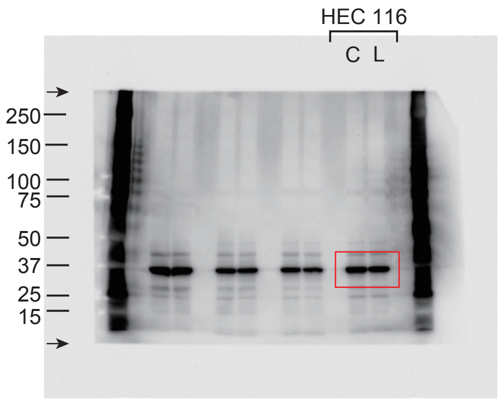

E. Western blot bands of LSR (i), MT1-MMP (ii), MMP2 (iii), and GAPDH (iv) after transfection of control-siRNA or LSR-siRNA in HEC1 cells (Original data of Figure 4D).

Red boxes denote the regions of original blots used in Figure 4D. Arrows indicate the edge of the membrane. Abbreviations: C, Control-siRNA; L, LSR-siRNA.

(i) LSR (65 kDa)

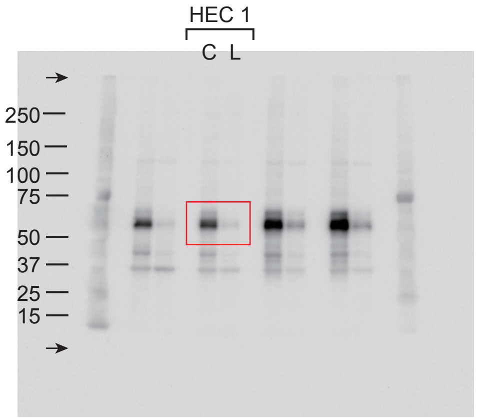

(ii) MT1-MMP (50,62 kDa)

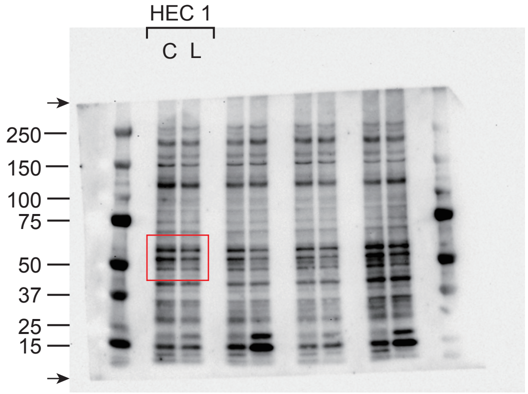

(iii) MMP2 (72 kDa)

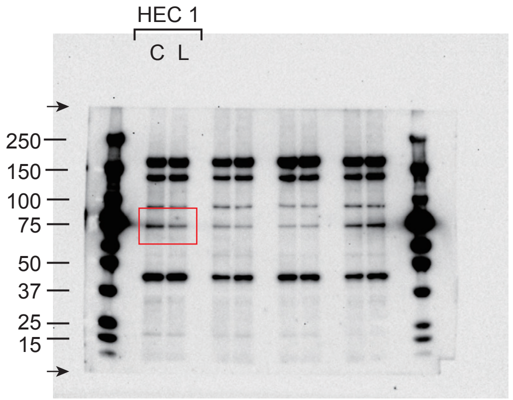

(iv) GAPDH (37 kDa)

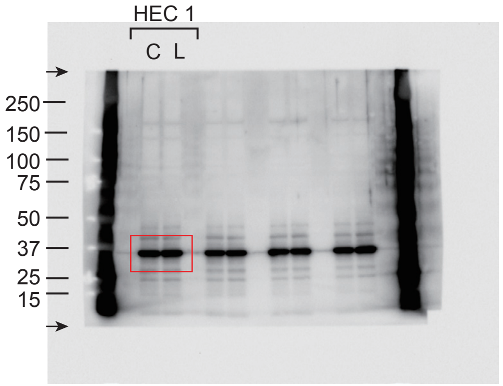

F. Western blot bands of LSR (i), MT1-MMP (ii), MMP2 (iii), and GAPDH (iv) after transfection of control-siRNA or LSR-siRNA in HEC116 cells (Original data of Figure 4D).

Red boxes denote the regions of original blots used in Figure 4D. Arrows indicate the edge of the membrane. Abbreviations: C, Control-siRNA; L, LSR-siRNA.

(i) LSR (65 kDa)

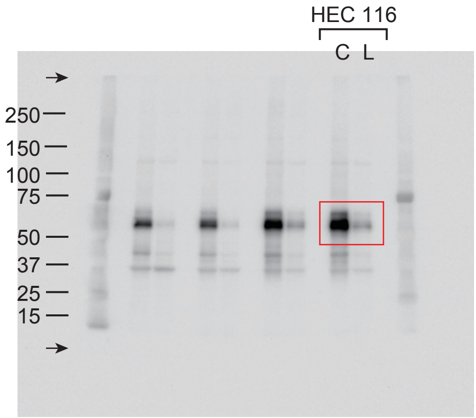

(ii) MT1-MMP (50,62 kDa)

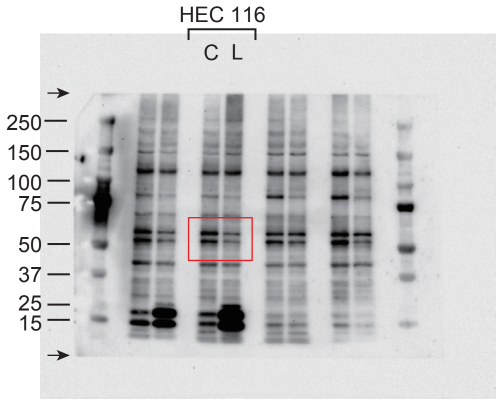

(iii) MMP2 (72 kDa)

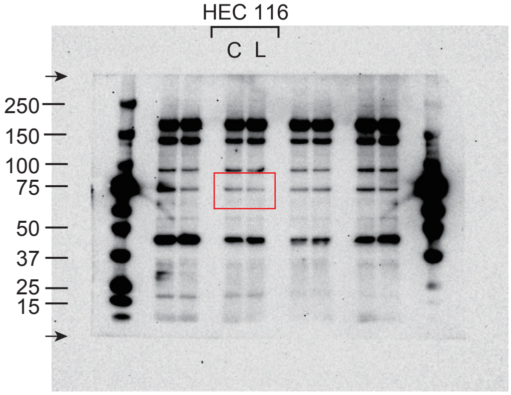

(iv) GAPDH (37 kDa)

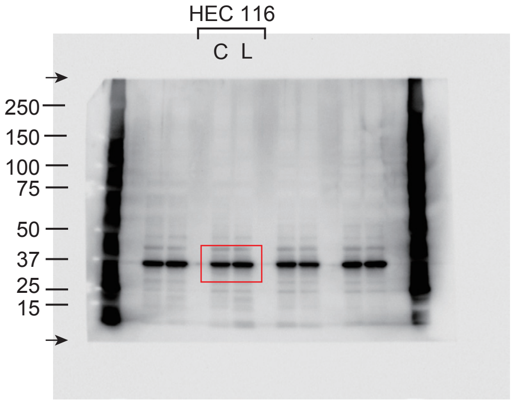

G. Western blot bands of LSR (i), phospho-ERK1/2 (ii), ERK1/2 (iii), MT1-MMP (iv), MMP2 (v), and GAPDH (vi) after transfection of control-siRNA or ERK1/2-siRNA in HEC1 cells (Original data of Figure 4G).

Red boxes denote the regions of original blots used in Figure 4G. Arrows indicate the edge of the membrane. Abbreviations: C, Control-siRNA; E, ERK1/2-siRNA.

(i) LSR (65 kDa)

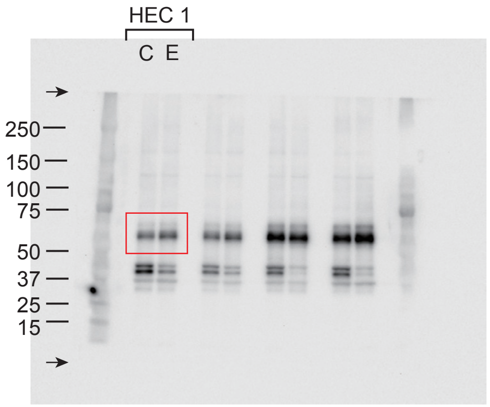

(ii) phospho-ERK1/2 (42,44 kDa)

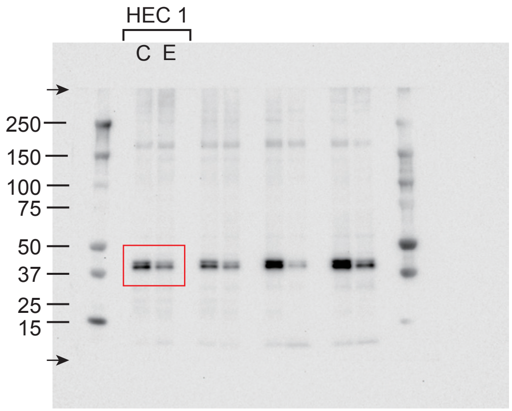

(iii) ERK1/2 (42,44 kDa)

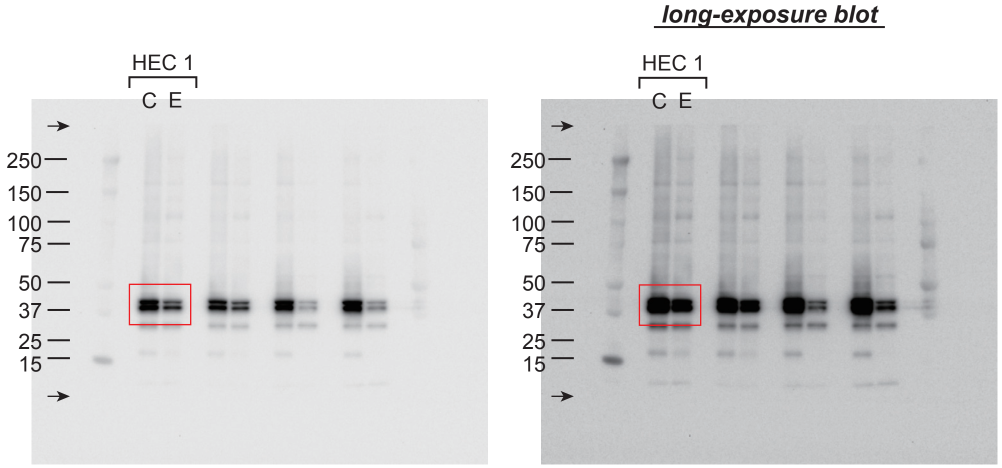

(iv) MT1-MMP (50,62 kDa)

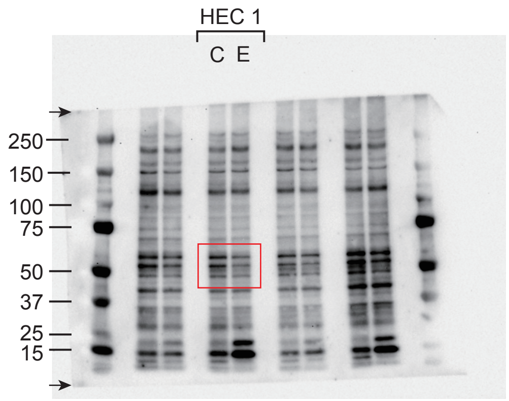

(v) MMP2 (72 kDa)

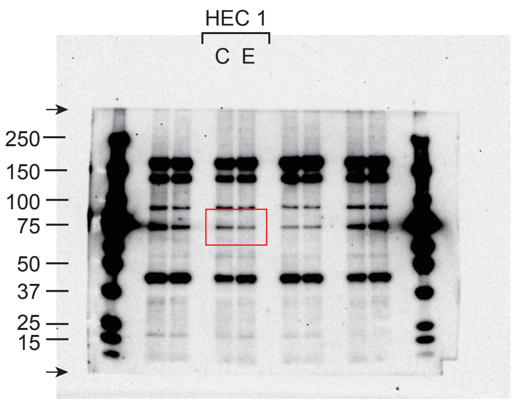

(vi) GAPDH (37 kDa)

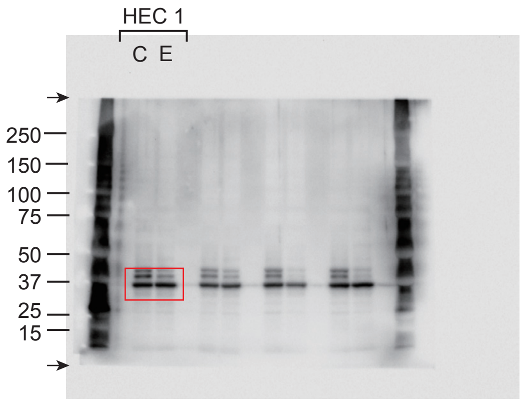

H. Western blot bands of LSR (i), phospho-ERK1/2 (ii), ERK1/2 (iii), MT1-MMP (iv), MMP2 (v), and GAPDH (vi) after transfection of control-siRNA or ERK1/2-siRNA in HEC116 cells (Original data of Figure 4G).

Red boxes denote the regions of original blots used in Figure 4G. Arrows indicate the edge of the membrane. Abbreviations: C, Control-siRNA; E, ERK1/2-siRNA.

(i) LSR (65 kDa)

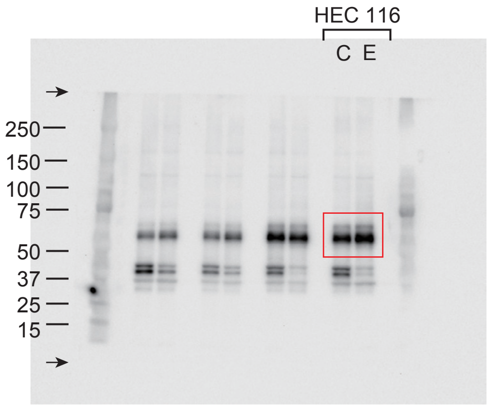

(ii) phospho-ERK1/2 (42,44 kDa)

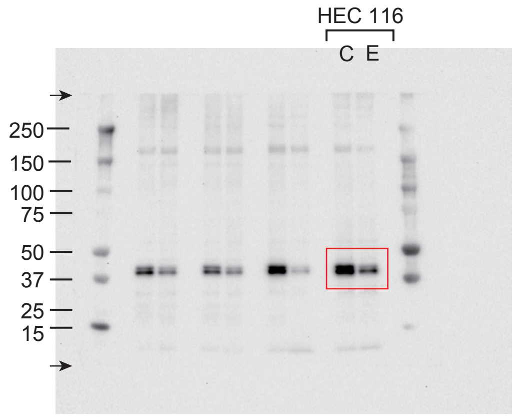

(iii) ERK1/2 (42,44 kDa)

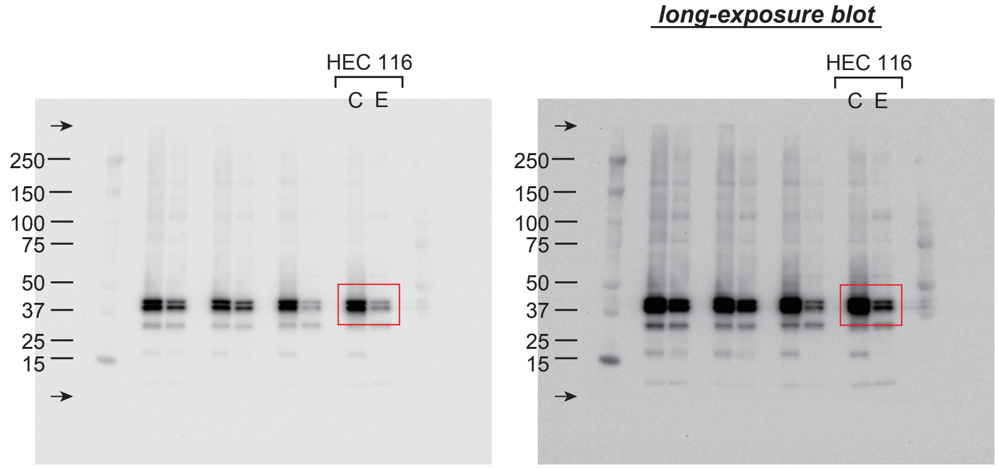

(iv) MT1-MMP (50,62 kDa)

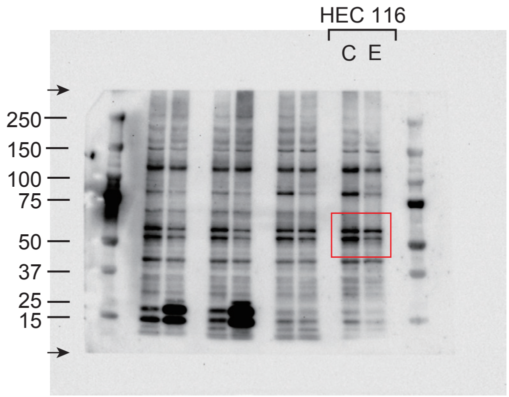

(v) MMP2 (72 kDa)

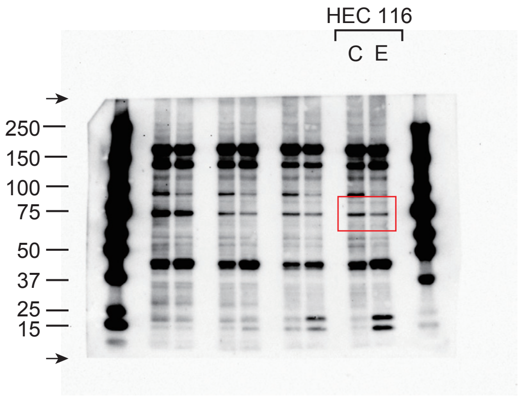

(vi) GAPDH (37 kDa)

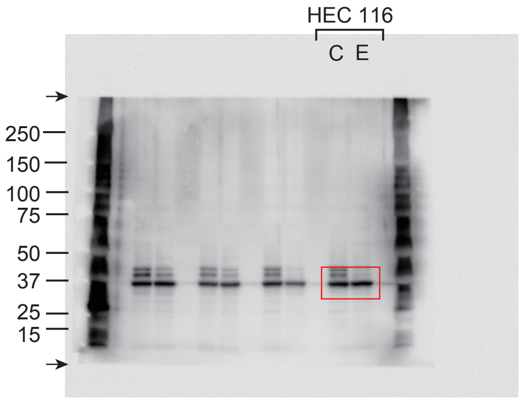

I. Western blot bands of LSR (i), phospho-SAPK/JNK (ii), SAPK/JNK (iii), phospho-p38 MAPK (iv), p38 MAPK (v), and GAPDH (vi) after transfection of control-siRNA or LSR-siRNA in HEC1 cells (Original data of Supplemental Figure S2).

Red boxes denote the regions of original blots used in Supplemental Figure S2. Arrows indicate the edge of the membrane. Abbreviations: C, Control-siRNA; L, LSR-siRNA.

(i) LSR (65 kDa)

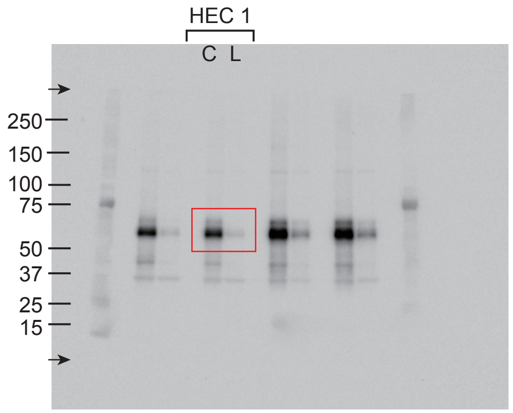

(ii) phospho-SAPK/JNK (48,54 kDa)

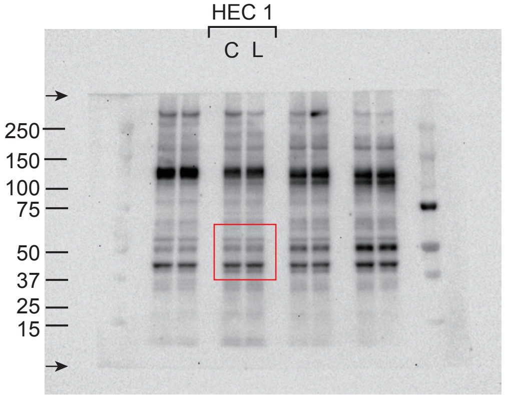

(iii) SAPK/JNK (48,54 kDa)

long-exposure blot

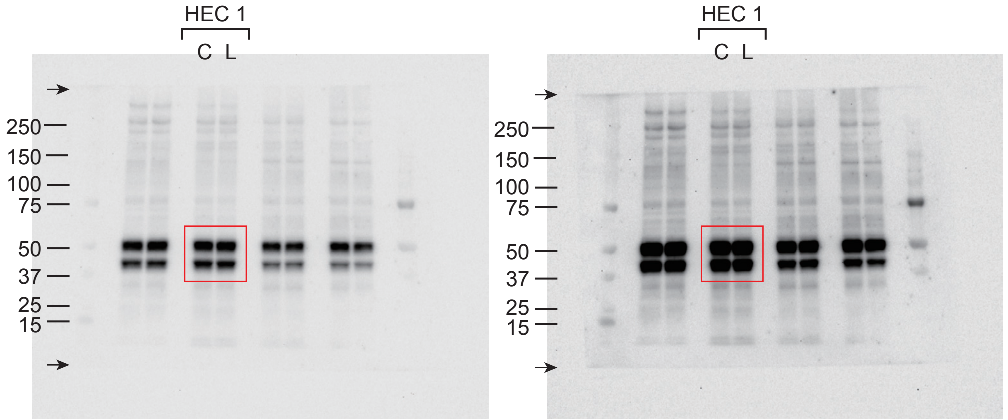

(iv) phospho-p38 MAPK (38 kDa)

long-exposure blot

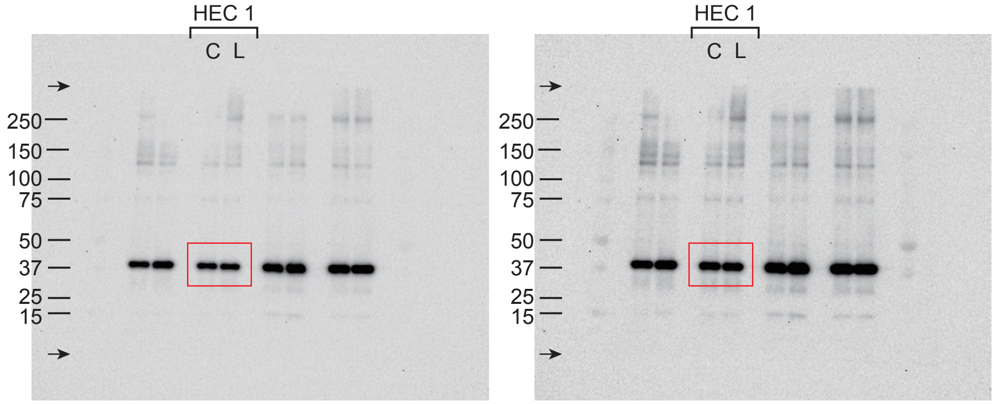

(v) p38 MAPK (38 kDa)

long-exposure blot

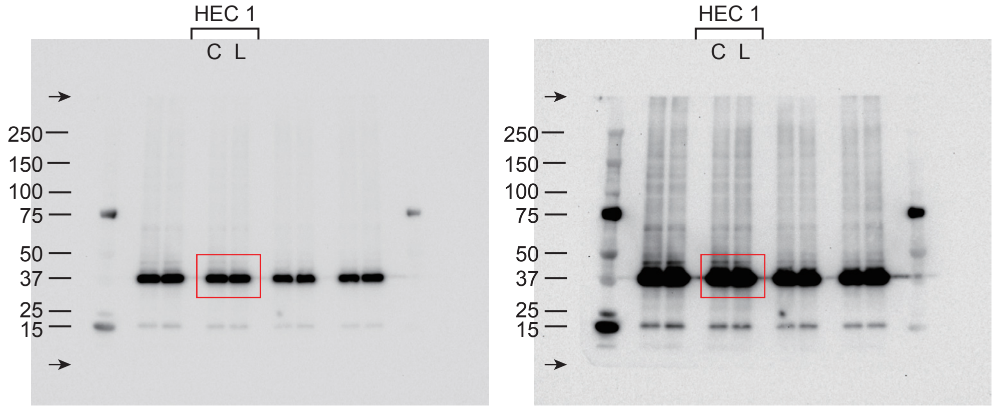

(vi) GAPDH (37 kDa)

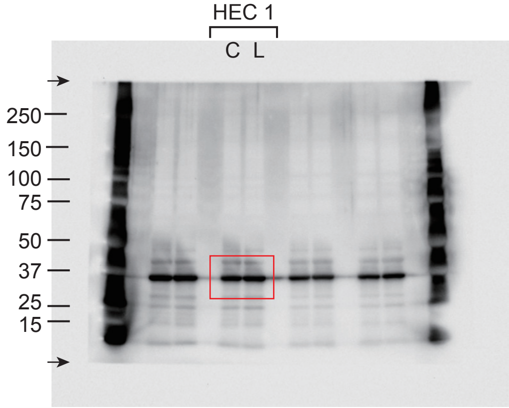

J. Western blot bands of LSR (i), phospho-SAPK/JNK (ii), SAPK/JNK (iii), phospho-p38 MAPK (iv), p38 MAPK (v), and GAPDH (vi) after transfection of control-siRNA or LSR-siRNA in HEC116 cells (Original data of Supplemental Figure S2).

Red boxes denote the regions of original blots used in Supplemental Figure S2. Arrows indicate the edge of the membrane. Abbreviations: C, Control-siRNA; L, LSR-siRNA.

(i) LSR (65 kDa)

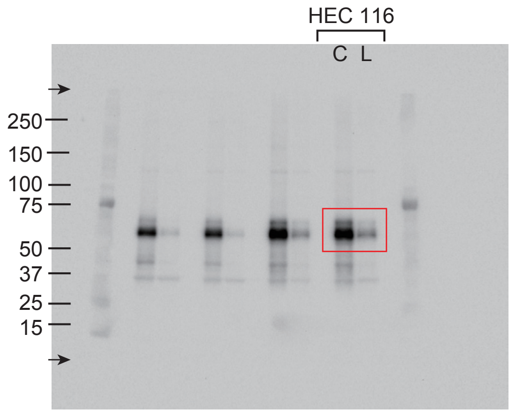

(ii) phospho-SAPK/JNK (48,54 kDa)

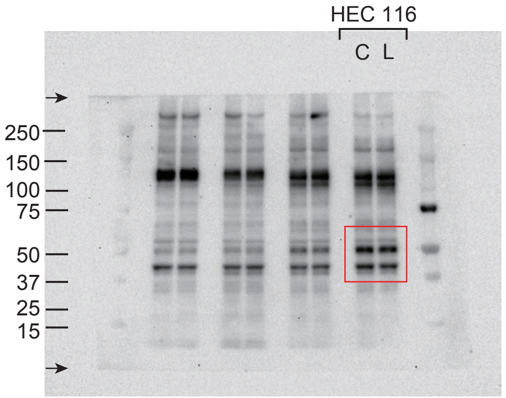

(iii) SAPK/JNK (48,54 kDa)

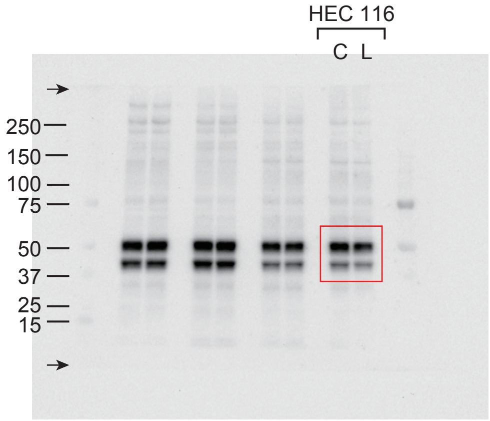

long-exposure blot

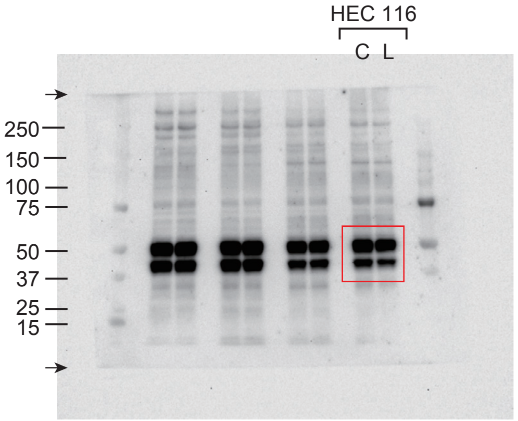

(iv) phospho-p38 MAPK (38 kDa)

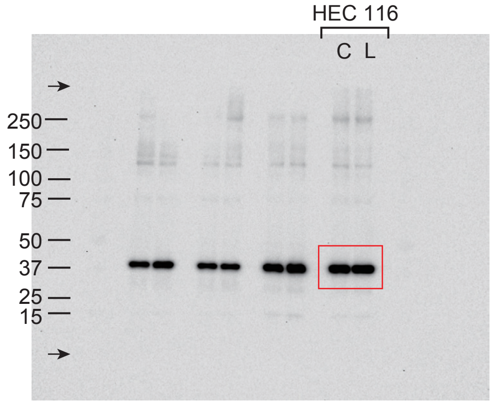

long-exposure blot

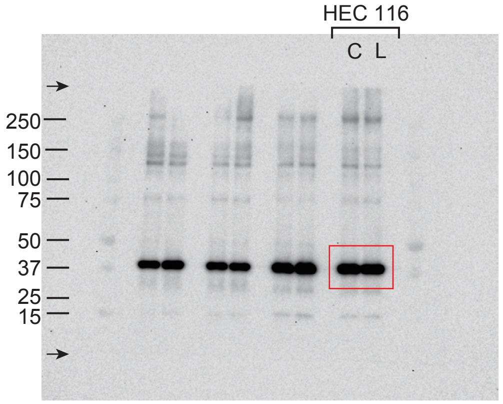

(v) p38 MAPK (38 kDa)

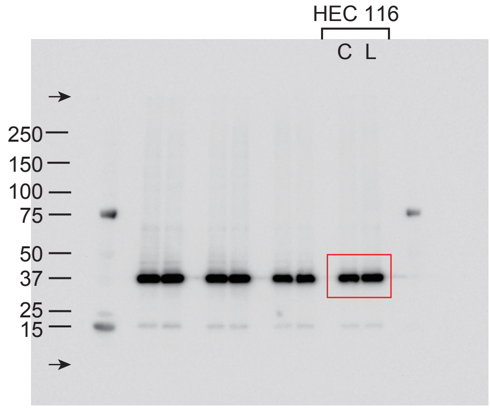

long-exposure blot

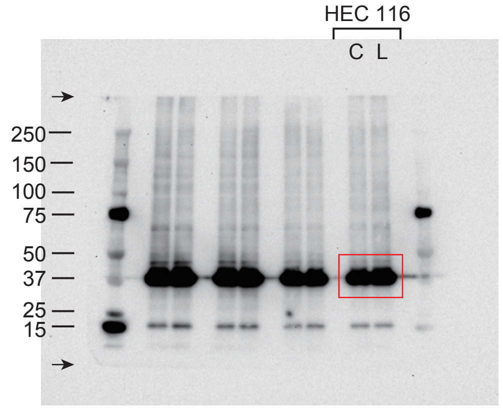

(vi) GAPDH (37 kDa)

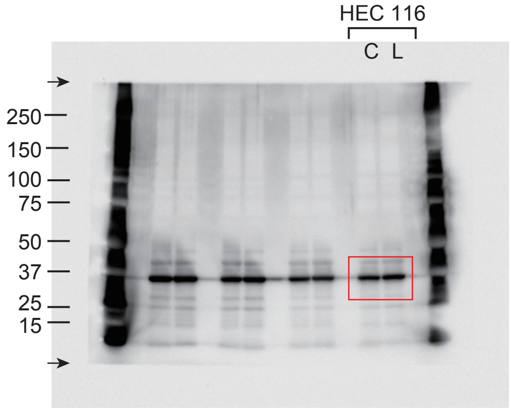

Supplement: Supplementary file 3 — Additional file 3. [file 12885_2022_9789_MOESM3_ESM.pdf]
