# Supplementary material for: Anti-lipolysis-stimulated lipoprotein receptor monoclonal antibody as a novel therapeutic agent for endometrial cancer
Source: BMC Cancer. 2022 Jun 21;22:679. doi: 10.1186/s12885-022-09789-6 (PMC9210735; doi:10.1186/s12885-022-09789-6)
Supplement: Supplementary file 4 — Additional file 4. [file 12885_2022_9789_MOESM4_ESM.docx]

**Supplemental method S1. Detailed and additional information of Methods.**

***Patients and tissue samples***

Formalin-fixed and paraffin-embedded tissue sections with a thickness of 4 μm were prepared from 228 patients with endometrial cancer (EC) who underwent a hysterectomy between 2006 and 2015. We retrospectively reviewed the following clinical characteristics and outcomes: (*i*) age, (*ii*) body mass index, (*iii*) histological subtype, (*iv*) stage (according to the 2008 International Federation of Gynecology and Obstetrics [FIGO] staging system [1]), (*v*) depth of uterine myometrial invasion, (*vi*) tumor involvement of the adnexa or serosa, (*vii*) lymphovascular space invasion, (*viii*) lymph node metastasis, (*ix*) distant metastasis, and (*x*) overall survival. Patients with relatively rare histological subtypes, such as clear cell and mucinous carcinoma, and patients who received preoperative radiation therapy or chemotherapy, were excluded from this study. This study was approved by the Osaka University Research Ethics Committee (Approval No. 19241, October 16, 2019). We obtained a written informed consent from all patients and conducted this study in accordance with the Declaration of Helsinki.

***Immunohistochemical analysis***

Immunohistochemistry (IHC) staining was performed using an anti-LSR antibody (#14804), anti-MMP2 antibody (#40994), and anti-phospho-ERK1/2 antibody (Thr202/Tyr204) (#4370) from Cell Signaling Technology (MA, USA) as previously described [2, 3]. Three gynecologic oncologists trained in pathological diagnosis individually evaluated the staining intensity and distribution of tumor tissue which was scored. IHC scoring was evaluated without being informed of the patient characteristics. In the event of disagreement, scoring was decided by a majority vote.

***Western blot analysis***

We used the following specific antibodies: anti-LSR (#14804), anti-phospho-MEK1/2 (Ser217/221) (#9154), anti-MEK1/2 (#9122), anti-phospho-ERK1/2 (Thr202/Tyr204) (#4370), anti-ERK1/2 (#4695), anti-phospho-p90RSK (Ser380) (#11989), anti-p90RSK (RSK1/RSK2/RSK3) (#9355), anti-phospho-SAPK/JNK (Thr183/Tyr185) (#4668), anti-SAPK/JNK (#9252), anti-phospho-p38 MAPK (Thr180/Tyr182) (#4511), anti-p38 MAPK (#8690), anti-MT1-MMP (#13130), and anti-MMP2 (#40994) from Cell Signaling Technology (MA, USA); and anti-GAPDH (sc-25778) from Santa Cruz Biotechnology (CA, USA).

***Cell invasion and migration assay***

Cell invasion assay was performed using CytoSelect cell invasion assay kit (CBA-112, Cell Biolabs, CA, USA) and cell migration assay was conducted using transwell chambers with an 8.0 μm pore (Corning, Falcon, NY, USA). HEC1 and HEC116 cells transfected with control-siRNA or LSR-siRNA were suspended in serum-free media in the upper chamber which had an 8.0 μm pore membrane. The lower chamber, outside of the membrane, was filled with media containing fetal bovine serum, and the cells were incubated at 37°C for 24 hours. After incubation, invading cells or migrating cells were analyzed.

***Pathway enrichment and ontology analysis***

Pathway enrichment and ontology analysis was performed using a published proteomic dataset for EC [4]. The Database for Annotation, Visualization, and Integrated Discovery (DAVID) was used to analyze the Kyoto Encyclopedia of Gene and Genome (KEGG) pathways and functional annotations [5, 6]. After excluding normal endometrial samples from this dataset, the expression of 10,999 proteins were re-standardized and analyzed in 95 EC samples for which clinical information was registered. Proteins with missing expression data were excluded from the analysis. A final set of 8,017 proteins were included in the analysis. In the high-LSR sample group, which included 24 EC samples with LSR expression levels in the upper quartile, the Pearson’s correlation coefficients were calculated for the expression of all proteins compared with LSR expression levels. Proteomic data for 873 proteins (10.9%) that correlated with LSR expression (correlation coefficient ≤−0.4 or ≥0.4) were analyzed using DAVID.

***Antibody therapy in xenograft mouse model***

Healthy female 6-week-old Institute of Cancer Research nu/nu mice were provided by Charles River Japan (Yokohama, Japan). For the development of a xenograft model, 2.0 × 10^6^ HEC1 cells in 100 μl of phosphate-buffered saline (PBS) and Matrigel (Corning, NY, USA) were injected subcutaneously. Tumor volumes were evaluated twice a week [tumor volume (mm^3^) = length (mm) × width (mm) × width (mm) × 0.5]. When the mean tumor volume reached approximately 100 mm^3^, the mice were randomized into two groups (5 mice per group) to receive antibody therapy. We used an isotype control mouse IgG2a antibody (control Ab) (Sigma Aldrich, MO, USA) or chimeric chicken–mouse anti-LSR monoclonal antibody (#1–25; Pharmafoods) that was described in our previous reports [2, 3]. These antibodies were administrated intraperitoneally at a dose of 200 μg/body in 400 μl PBS twice a week for 3 weeks. The mice were sacrificed 4 weeks after initiating the treatment.

**Supplemental References**

1. Pecorelli S: **Revised FIGO staging for carcinoma of the vulva, cervix, and endometrium**. *Int J Gynaecol Obstet* 2009, **105**(2):103-104.

2. Hiramatsu K, Serada S, Enomoto T, Takahashi Y, Nakagawa S, Nojima S, Morimoto A, Matsuzaki S, Yokoyama T, Takahashi T *et al*: **LSR Antibody Therapy Inhibits Ovarian Epithelial Tumor Growth by Inhibiting Lipid Uptake**. *Cancer Res* 2018, **78**(2):516-527.

3. Sugase T, Takahashi T, Serada S, Fujimoto M, Ohkawara T, Hiramatsu K, Koh M, Saito Y, Tanaka K, Miyazaki Y *et al*: **Lipolysis-stimulated lipoprotein receptor overexpression is a novel predictor of poor clinical prognosis and a potential therapeutic target in gastric cancer**. *Oncotarget* 2018, **9**(68):32917-32928.

4. Dou Y, Kawaler EA, Cui Zhou D, Gritsenko MA, Huang C, Blumenberg L, Karpova A, Petyuk VA, Savage SR, Satpathy S *et al*: **Proteogenomic Characterization of Endometrial Carcinoma**. *Cell* 2020, **180**(4):729-748.e726.

5. Kanehisa M, Goto S: **KEGG: kyoto encyclopedia of genes and genomes**. *Nucleic Acids Res* 2000, **28**(1):27-30.

6. Bielenberg GW, Beck T: **The effects of dizocilpine (MK-801), phencyclidine, and nimodipine on infarct size 48 h after middle cerebral artery occlusion in the rat**. *Brain Res* 1991, **552**(2):338-342.
