# Supplementary material for: Anti-lipolysis-stimulated lipoprotein receptor monoclonal antibody as a novel therapeutic agent for endometrial cancer
Source: BMC Cancer. 2022 Jun 21;22:679. doi: 10.1186/s12885-022-09789-6 (PMC9210735; doi:10.1186/s12885-022-09789-6)
Supplement: Supplementary file 5 — Additional file 5. [file 12885_2022_9789_MOESM5_ESM.docx]

**Supplemental Table S1. Multivariate analysis to identify factors associated with poor prognosis in endometrial cancer.**

|  | Univariate analysis | | |  | Multivariate analysis | | |
| --- | --- | --- | --- | --- | --- | --- | --- |
| Variables | Crude HR | 95% CI | p value |  | Adjusted HR | 95% CI | p value |
| LSR expression  [High / Low] | 3.53 | 1.35 – 9.24 | 0.01 |  | 2.07 | 0.76 – 5.61 | 0.15 |
| Histological grade  [High grade / Low grade] | 3.53 | 1.68 – 7.40 | < 0.01 |  | 1.95 | 0.89 – 4.24 | 0.094 |
| Tumor involvement of adnexa or serosa  [Positive / Negative] | 5.85 | 3.0 – 11.4 | < 0.01 |  | 0.97 | 0.39 – 2.39 | 0.94 |
| Lymph node metastasis  [Positive / Negative] | 5.25 | 2.67 – 10.3 | < 0.01 |  | 0.92 | 0.37 – 2.33 | 0.87 |
| Distant metastasis  [Positive / Negative] | 13.1 | 6.59 – 26.1 | < 0.01 |  | 4.49 | 1.93 – 10.5 | < 0.01 |
| FIGO Stage  [III-IV / I-II] | 8.01 | 3.63 – 17.7 | < 0.01 |  | 3.84 | 1.11 – 13.3 | 0.034 |

Abbreviations: HR, hazard ratio; CI, confidence interval; LSR, Lipolysis-stimulated lipoprotein receptor; and FIGO, the International Federation of Gynecology and Obstetrics.
