# Supplementary material for: Anti-lipolysis-stimulated lipoprotein receptor monoclonal antibody as a novel therapeutic agent for endometrial cancer
Source: BMC Cancer. 2022 Jun 21;22:679. doi: 10.1186/s12885-022-09789-6 (PMC9210735; doi:10.1186/s12885-022-09789-6)
Supplement: Supplementary file 6 — Additional file 6. [file 12885_2022_9789_MOESM6_ESM.docx]

**Supplemental Table S2. Pathway enrichment analysis of proteins correlating with high-LSR expression using published proteomic data of endometrial cancer.**

| Entry | | Name | Count | % | p value | Benjamini q-value |
| --- | --- | --- | --- | --- | --- | --- |
| ***Environmental Information Processing - Signal transduction*** | | | | | | |
|  | map04010 | MAPK signaling pathway | 19 | 2.2 | 0.091 | 0.66 |
|  | map04330 | Notch signaling pathway | 6 | 0.7 | 0.094 | 0.66 |
| ***Genetic Information Processing - Folding, sorting and degradation*** | | | | | | |
|  | map04120 | Ubiquitin mediated proteolysis | 14 | 1.6 | 0.021 | 0.49 |
|  | map03050 | Proteasome | 10 | 1.1 | 0.00029 | 0.036 |
|  | map04130 | SNARE interactions in vesicular transport | 9 | 1.0 | 0.00023 | 0.056 |
|  | map04122 | Sulfur relay system | 3 | 0.3 | 0.088 | 0.69 |
| ***Metabolism*** | | | | | | |
|  | map01100 | Metabolic pathways | 84 | 9.6 | 0.002 | 0.12 |
|  | map00980 | Metabolism of xenobiotics by cytochrome P450 | 11 | 1.3 | 0.0039 | 0.18 |
|  | map01059 | Biosynthesis of antibiotics | 19 | 2.2 | 0.022 | 0.46 |
|  | map00982 | Drug metabolism – cytochrome P450 | 8 | 0.9 | 0.056 | 0.57 |
|  | map00380 | Tryptophan metabolism | 7 | 0.8 | 0.015 | 0.41 |
|  | map00280 | Valine, leucine and isoleucine degradation | 7 | 0.8 | 0.03 | 0.48 |
|  | map00480 | Glutathione metabolism | 7 | 0.8 | 0.043 | 0.5 |
|  | map00250 | Alanine, aspartate and glutamate metabolism | 6 | 0.7 | 0.03 | 0.45 |
|  | map00270 | Cysteine and methionine metabolism | 6 | 0.7 | 0.041 | 0.51 |
|  | map00900 | Terpenoid backbone biosynthesis | 5 | 0.6 | 0.023 | 0.44 |
|  | map00760 | Nicotinate and nicotinamide metabolism | 5 | 0.6 | 0.057 | 0.56 |
|  | map00100 | Steroid biosynthesis | 4 | 0.5 | 0.078 | 0.66 |
| ***Cellular Processes - Transport and catabolism*** | | | | | | |
|  | map04146 | Peroxisome | 10 | 1.1 | 0.024 | 0.43 |
| ***Organismal Systems*** | | | | | | |
|  | map04721 | Synaptic vesicle cycle | 8 | 0.9 | 0.039 | 0.51 |
|  | map04612 | Antigen processing and presentation | 8 | 0.9 | 0.09 | 0.68 |
| ***Human Diseases*** | | | | | | |
|  | map05169 | Epstein-Barr virus infection | 17 | 1.9 | 0.00041 | 0.034 |
|  | map05204 | Chemical carcinogenesis | 11 | 1.3 | 0.0069 | 0.25 |

Bold italics indicate the classification in the Kyoto Encyclopedia of Gene and Genome pathway.
