# Supplementary material for: Anti-lipolysis-stimulated lipoprotein receptor monoclonal antibody as a novel therapeutic agent for endometrial cancer
Source: BMC Cancer. 2022 Jun 21;22:679. doi: 10.1186/s12885-022-09789-6 (PMC9210735; doi:10.1186/s12885-022-09789-6)
Supplement: Supplementary file 7 — Additional file 7. [file 12885_2022_9789_MOESM7_ESM.docx]

**Supplemental Table S3. Ontology analysis of proteins correlating with high-LSR expression using published proteomic data of endometrial cancer.**

| Entry | Term | Count | % | p value | Benjamini  q value |
| --- | --- | --- | --- | --- | --- |
| GO:0060271 | cilium morphogenesis | 34 | 3.9 | 3.2E-15 | 1.0E-11 |
| GO:0042384 | cilium assembly | 32 | 3.7 | 9.1E-15 | 1.5E-11 |
| GO:0000226 | microtubule cytoskeleton organization | 15 | 1.7 | 4.5E-06 | 4.8E-03 |
| GO:0061512 | protein localization to cilium | 8 | 0.9 | 9.9E-06 | 8.0E-03 |
| GO:0038061 | NIK/NF-kappaB signaling | 14 | 1.6 | 9.9E-06 | 6.4E-03 |
| GO:0035721 | intraciliary retrograde transport | 6 | 0.7 | 4.6E-05 | 2.5E-02 |
| GO:0042073 | intraciliary transport | 7 | 0.8 | 5.6E-05 | 2.6E-02 |
| GO:0006521 | regulation of cellular amino acid metabolic process | 11 | 1.3 | 1.1E-04 | 4.3E-02 |
| GO:0090263 | positive regulation of canonical Wnt signaling pathway | 17 | 1.9 | 1.5E-04 | 5.4E-02 |
| GO:0090090 | negative regulation of canonical Wnt signaling pathway | 20 | 2.3 | 2.4E-04 | 7.3E-02 |
| GO:0006886 | intracellular protein transport | 25 | 2.9 | 3.2E-04 | 8.9E-02 |
| GO:0002223 | stimulatory C-type lectin receptor signaling pathway | 15 | 1.7 | 3.9E-04 | 1.0E-01 |
| GO:0043124 | negative regulation of I-kappaB kinase/NF-kappaB signaling | 9 | 1.0 | 4.6E-04 | 1.1E-01 |
| GO:0007266 | Rho protein signal transduction | 10 | 1.1 | 4.7E-04 | 1.0E-01 |
| GO:0048278 | vesicle docking | 7 | 0.8 | 5.3E-04 | 1.1E-01 |
| GO:0002479 | antigen processing and presentation of exogenous peptide antigen via MHC class I, TAP-dependent | 11 | 1.3 | 6.5E-04 | 1.2E-01 |
| GO:0006897 | endocytosis | 17 | 1.9 | 8.2E-04 | 1.4E-01 |
| GO:0000209 | protein polyubiquitination | 20 | 2.3 | 1.1E-03 | 1.7E-01 |
| GO:0060071 | Wnt signaling pathway, planar cell polarity pathway | 13 | 1.5 | 1.2E-03 | 1.9E-01 |
| GO:0007224 | smoothened signaling pathway | 11 | 1.3 | 1.4E-03 | 2.0E-01 |
| GO:0006914 | autophagy | 16 | 1.8 | 1.4E-03 | 2.0E-01 |
| GO:1902017 | regulation of cilium assembly | 8 | 0.9 | 1.5E-03 | 1.9E-01 |
| GO:0050852 | T cell receptor signaling pathway | 17 | 1.9 | 1.6E-03 | 2.0E-01 |
| GO:0051436 | negative regulation of ubiquitin-protein ligase activity involved in mitotic cell cycle | 11 | 1.3 | 1.7E-03 | 2.0E-01 |
| GO:0006695 | cholesterol biosynthetic process | 8 | 0.9 | 1.7E-03 | 2.0E-01 |
| GO:0006541 | glutamine metabolic process | 6 | 0.7 | 1.9E-03 | 2.1E-01 |
| GO:0043065 | positive regulation of apoptotic process | 27 | 3.1 | 2.0E-03 | 2.1E-01 |
| GO:0001947 | heart looping | 10 | 1.1 | 2.0E-03 | 2.1E-01 |
| GO:0032880 | regulation of protein localization | 9 | 1.0 | 2.7E-03 | 2.6E-01 |
| GO:0051437 | positive regulation of ubiquitin-protein ligase activity involved in regulation of mitotic cell cycle transition | 11 | 1.3 | 2.8E-03 | 2.6E-01 |
| GO:0043066 | negative regulation of apoptotic process | 36 | 4.1 | 2.9E-03 | 2.6E-01 |
| GO:0032465 | regulation of cytokinesis | 6 | 0.7 | 3.0E-03 | 2.7E-01 |
| GO:1901687 | glutathione derivative biosynthetic process | 6 | 0.7 | 3.0E-03 | 2.7E-01 |
| GO:0035735 | intraciliary transport involved in cilium morphogenesis | 4 | 0.5 | 3.1E-03 | 2.6E-01 |
| GO:0043488 | regulation of mRNA stability | 13 | 1.5 | 3.2E-03 | 2.6E-01 |
| GO:0031145 | anaphase-promoting complex-dependent catabolic process | 11 | 1.3 | 3.8E-03 | 2.9E-01 |
| GO:0045880 | positive regulation of smoothened signaling pathway | 6 | 0.7 | 4.5E-03 | 3.4E-01 |
| GO:0030336 | negative regulation of cell migration | 12 | 1.4 | 4.9E-03 | 3.5E-01 |
| GO:0032091 | negative regulation of protein binding | 9 | 1.0 | 4.9E-03 | 3.4E-01 |
| GO:0071902 | positive regulation of protein serine/threonine kinase activity | 7 | 0.8 | 5.3E-03 | 3.6E-01 |
| GO:0070373 | negative regulation of ERK1 and ERK2 cascade | 9 | 1.0 | 5.4E-03 | 3.6E-01 |
| GO:0008152 | metabolic process | 17 | 1.9 | 5.7E-03 | 3.6E-01 |
| GO:0009636 | response to toxic substance | 11 | 1.3 | 6.4E-03 | 3.9E-01 |
| GO:0019287 | isopentenyl diphosphate biosynthetic process, mevalonate pathway | 3 | 0.3 | 6.4E-03 | 3.8E-01 |
| GO:0061077 | chaperone-mediated protein folding | 7 | 0.8 | 7.0E-03 | 4.0E-01 |
| GO:0035058 | nonmotile primary cilium assembly | 6 | 0.7 | 7.7E-03 | 4.3E-01 |
| GO:2001237 | negative regulation of extrinsic apoptotic signaling pathway | 7 | 0.8 | 8.0E-03 | 4.3E-01 |
| GO:0007020 | microtubule nucleation | 5 | 0.6 | 8.7E-03 | 4.5E-01 |
| GO:0033209 | tumor necrosis factor-mediated signaling pathway | 13 | 1.5 | 9.4E-03 | 4.7E-01 |
| GO:0007286 | spermatid development | 10 | 1.1 | 9.8E-03 | 4.8E-01 |
| GO:0006631 | fatty acid metabolic process | 8 | 0.9 | 1.0E-02 | 4.9E-01 |
| GO:0043393 | regulation of protein binding | 5 | 0.6 | 1.1E-02 | 4.9E-01 |
| GO:0019886 | antigen processing and presentation of exogenous peptide antigen via MHC class II | 11 | 1.3 | 1.1E-02 | 5.0E-01 |
| GO:0015031 | protein transport | 30 | 3.4 | 1.2E-02 | 5.1E-01 |
| GO:0032480 | negative regulation of type I interferon production | 6 | 0.7 | 1.2E-02 | 5.2E-01 |
| GO:0006760 | folic acid-containing compound metabolic process | 3 | 0.3 | 1.2E-02 | 5.2E-01 |
| GO:0006081 | cellular aldehyde metabolic process | 4 | 0.5 | 1.3E-02 | 5.3E-01 |
| GO:0090002 | establishment of protein localization to plasma membrane | 7 | 0.8 | 1.3E-02 | 5.2E-01 |
| GO:0006979 | response to oxidative stress | 12 | 1.4 | 1.4E-02 | 5.5E-01 |
| GO:0000413 | protein peptidyl-prolyl isomerization | 7 | 0.8 | 1.5E-02 | 5.5E-01 |
| GO:0043161 | proteasome-mediated ubiquitin-dependent protein catabolic process | 18 | 2.1 | 1.5E-02 | 5.6E-01 |
| GO:0050680 | negative regulation of epithelial cell proliferation | 8 | 0.9 | 1.5E-02 | 5.6E-01 |
| GO:0061024 | membrane organization | 6 | 0.7 | 1.6E-02 | 5.6E-01 |
| GO:0043547 | positive regulation of GTPase activity | 39 | 4.5 | 1.7E-02 | 5.8E-01 |
| GO:0030307 | positive regulation of cell growth | 10 | 1.1 | 1.7E-02 | 5.8E-01 |
| GO:0031334 | positive regulation of protein complex assembly | 5 | 0.6 | 1.8E-02 | 5.9E-01 |
| GO:0045736 | negative regulation of cyclin-dependent protein serine/threonine kinase activity | 5 | 0.6 | 1.8E-02 | 5.9E-01 |
| GO:2000467 | positive regulation of glycogen (starch) synthase activity | 3 | 0.3 | 2.0E-02 | 6.3E-01 |
| GO:0038095 | Fc-epsilon receptor signaling pathway | 16 | 1.8 | 2.1E-02 | 6.3E-01 |
| GO:0051492 | regulation of stress fiber assembly | 4 | 0.5 | 2.1E-02 | 6.3E-01 |
| GO:0009168 | purine ribonucleoside monophosphate biosynthetic process | 4 | 0.5 | 2.1E-02 | 6.3E-01 |
| GO:0001967 | suckling behavior | 4 | 0.5 | 2.1E-02 | 6.3E-01 |
| GO:0090398 | cellular senescence | 5 | 0.6 | 2.1E-02 | 6.3E-01 |
| GO:0030433 | ER-associated ubiquitin-dependent protein catabolic process | 8 | 0.9 | 2.2E-02 | 6.4E-01 |
| GO:0051289 | protein homotetramerization | 8 | 0.9 | 2.2E-02 | 6.4E-01 |
| GO:0000910 | cytokinesis | 7 | 0.8 | 2.4E-02 | 6.7E-01 |
| GO:0043409 | negative regulation of MAPK cascade | 4 | 0.5 | 2.5E-02 | 6.9E-01 |
| GO:0043508 | negative regulation of JUN kinase activity | 4 | 0.5 | 2.5E-02 | 6.9E-01 |
| GO:0021591 | ventricular system development | 4 | 0.5 | 2.5E-02 | 6.9E-01 |
| GO:0008299 | isoprenoid biosynthetic process | 4 | 0.5 | 2.5E-02 | 6.9E-01 |
| GO:0001516 | prostaglandin biosynthetic process | 4 | 0.5 | 2.5E-02 | 6.9E-01 |
| GO:0051091 | positive regulation of sequence-specific DNA binding transcription factor activity | 11 | 1.3 | 2.6E-02 | 6.8E-01 |
| GO:0030324 | lung development | 9 | 1.0 | 2.6E-02 | 6.8E-01 |
| GO:0050821 | protein stabilization | 13 | 1.5 | 2.6E-02 | 6.8E-01 |
| GO:0000086 | G2/M transition of mitotic cell cycle | 13 | 1.5 | 2.8E-02 | 7.0E-01 |
| GO:1990000 | amyloid fibril formation | 3 | 0.3 | 2.9E-02 | 7.1E-01 |
| GO:0007253 | cytoplasmic sequestering of NF-kappaB | 3 | 0.3 | 2.9E-02 | 7.1E-01 |
| GO:0071474 | cellular hyperosmotic response | 3 | 0.3 | 2.9E-02 | 7.1E-01 |
| GO:0071636 | positive regulation of transforming growth factor beta production | 3 | 0.3 | 2.9E-02 | 7.1E-01 |
| GO:0060296 | regulation of cilium beat frequency involved in ciliary motility | 3 | 0.3 | 2.9E-02 | 7.1E-01 |
| GO:0001701 | in utero embryonic development | 16 | 1.8 | 3.0E-02 | 7.2E-01 |
| GO:0006450 | regulation of translational fidelity | 4 | 0.5 | 3.1E-02 | 7.2E-01 |
| GO:1904874 | positive regulation of telomerase RNA localization to Cajal body | 4 | 0.5 | 3.1E-02 | 7.2E-01 |
| GO:0008344 | adult locomotory behavior | 7 | 0.8 | 3.4E-02 | 7.5E-01 |
| GO:1901800 | positive regulation of proteasomal protein catabolic process | 4 | 0.5 | 3.7E-02 | 7.7E-01 |
| GO:0030326 | embryonic limb morphogenesis | 6 | 0.7 | 3.8E-02 | 7.8E-01 |
| GO:0043312 | neutrophil degranulation | 3 | 0.3 | 3.9E-02 | 7.9E-01 |
| GO:0040012 | regulation of locomotion | 3 | 0.3 | 3.9E-02 | 7.9E-01 |
| GO:0010642 | negative regulation of platelet-derived growth factor receptor signaling pathway | 3 | 0.3 | 3.9E-02 | 7.9E-01 |
| GO:0003374 | dynamin polymerization involved in mitochondrial fission | 3 | 0.3 | 3.9E-02 | 7.9E-01 |
| GO:0051301 | cell division | 25 | 2.9 | 4.1E-02 | 8.0E-01 |
| GO:0090201 | negative regulation of release of cytochrome c from mitochondria | 4 | 0.5 | 4.3E-02 | 8.1E-01 |
| GO:0031333 | negative regulation of protein complex assembly | 4 | 0.5 | 4.3E-02 | 8.1E-01 |
| GO:2000785 | regulation of autophagosome assembly | 4 | 0.5 | 4.3E-02 | 8.1E-01 |
| GO:0021522 | spinal cord motor neuron differentiation | 4 | 0.5 | 4.3E-02 | 8.1E-01 |
| GO:0007368 | determination of left/right symmetry | 7 | 0.8 | 4.3E-02 | 8.1E-01 |
| GO:0060548 | negative regulation of cell death | 7 | 0.8 | 4.3E-02 | 8.1E-01 |
| GO:0006457 | protein folding | 15 | 1.7 | 4.4E-02 | 8.2E-01 |
| GO:0007163 | establishment or maintenance of cell polarity | 5 | 0.6 | 4.5E-02 | 8.2E-01 |
| GO:0016310 | phosphorylation | 10 | 1.1 | 4.5E-02 | 8.1E-01 |
| GO:0033138 | positive regulation of peptidyl-serine phosphorylation | 8 | 0.9 | 4.5E-02 | 8.1E-01 |
| GO:0098869 | cellular oxidant detoxification | 8 | 0.9 | 4.5E-02 | 8.1E-01 |
| GO:0035338 | long-chain fatty-acyl-CoA biosynthetic process | 6 | 0.7 | 4.6E-02 | 8.1E-01 |
| GO:0097191 | extrinsic apoptotic signaling pathway | 6 | 0.7 | 4.6E-02 | 8.1E-01 |
| GO:0021766 | hippocampus development | 7 | 0.8 | 4.7E-02 | 8.1E-01 |
| GO:0021987 | cerebral cortex development | 7 | 0.8 | 4.7E-02 | 8.1E-01 |
| GO:0070266 | necroptotic process | 4 | 0.5 | 5.0E-02 | 8.3E-01 |
| GO:0014823 | response to activity | 6 | 0.7 | 5.0E-02 | 8.3E-01 |
| GO:0070301 | cellular response to hydrogen peroxide | 7 | 0.8 | 5.0E-02 | 8.3E-01 |
| GO:0045773 | positive regulation of axon extension | 5 | 0.6 | 5.0E-02 | 8.2E-01 |
| GO:0045739 | positive regulation of DNA repair | 5 | 0.6 | 5.0E-02 | 8.2E-01 |
| GO:0021915 | neural tube development | 5 | 0.6 | 5.0E-02 | 8.2E-01 |
| GO:0001841 | neural tube formation | 3 | 0.3 | 5.1E-02 | 8.3E-01 |
| GO:0032688 | negative regulation of interferon-beta production | 3 | 0.3 | 5.1E-02 | 8.3E-01 |
| GO:0015939 | pantothenate metabolic process | 3 | 0.3 | 5.1E-02 | 8.3E-01 |
| GO:0060972 | left/right pattern formation | 3 | 0.3 | 5.1E-02 | 8.3E-01 |
| GO:0042987 | amyloid precursor protein catabolic process | 3 | 0.3 | 5.1E-02 | 8.3E-01 |
| GO:0044351 | macropinocytosis | 3 | 0.3 | 5.1E-02 | 8.3E-01 |
| GO:0016485 | protein processing | 8 | 0.9 | 5.2E-02 | 8.3E-01 |
| GO:0098609 | cell-cell adhesion | 20 | 2.3 | 5.3E-02 | 8.3E-01 |
| GO:0071392 | cellular response to estradiol stimulus | 5 | 0.6 | 5.5E-02 | 8.4E-01 |
| GO:0046827 | positive regulation of protein export from nucleus | 4 | 0.5 | 5.7E-02 | 8.5E-01 |
| GO:0006892 | post-Golgi vesicle-mediated transport | 4 | 0.5 | 5.7E-02 | 8.5E-01 |
| GO:0007026 | negative regulation of microtubule depolymerization | 4 | 0.5 | 5.7E-02 | 8.5E-01 |
| GO:0030220 | platelet formation | 4 | 0.5 | 5.7E-02 | 8.5E-01 |
| GO:0007338 | single fertilization | 7 | 0.8 | 5.8E-02 | 8.5E-01 |
| GO:0006906 | vesicle fusion | 7 | 0.8 | 5.8E-02 | 8.5E-01 |
| GO:0010629 | negative regulation of gene expression | 12 | 1.4 | 5.8E-02 | 8.5E-01 |
| GO:0070830 | bicellular tight junction assembly | 5 | 0.6 | 6.1E-02 | 8.6E-01 |
| GO:0007032 | endosome organization | 5 | 0.6 | 6.1E-02 | 8.6E-01 |
| GO:0000186 | activation of MAPKK activity | 6 | 0.7 | 6.3E-02 | 8.7E-01 |
| GO:0010595 | positive regulation of endothelial cell migration | 6 | 0.7 | 6.3E-02 | 8.7E-01 |
| GO:0045199 | maintenance of epithelial cell apical/basal polarity | 3 | 0.3 | 6.4E-02 | 8.7E-01 |
| GO:0010863 | positive regulation of phospholipase C activity | 3 | 0.3 | 6.4E-02 | 8.7E-01 |
| GO:0045899 | positive regulation of RNA polymerase II transcriptional preinitiation complex assembly | 3 | 0.3 | 6.4E-02 | 8.7E-01 |
| GO:2000786 | positive regulation of autophagosome assembly | 3 | 0.3 | 6.4E-02 | 8.7E-01 |
| GO:0035845 | photoreceptor cell outer segment organization | 3 | 0.3 | 6.4E-02 | 8.7E-01 |
| GO:0032886 | regulation of microtubule-based process | 3 | 0.3 | 6.4E-02 | 8.7E-01 |
| GO:0006972 | hyperosmotic response | 3 | 0.3 | 6.4E-02 | 8.7E-01 |
| GO:0032355 | response to estradiol | 9 | 1.0 | 6.4E-02 | 8.6E-01 |
| GO:2000377 | regulation of reactive oxygen species metabolic process | 4 | 0.5 | 6.5E-02 | 8.7E-01 |
| GO:0070059 | intrinsic apoptotic signaling pathway in response to endoplasmic reticulum stress | 5 | 0.6 | 6.7E-02 | 8.7E-01 |
| GO:0070555 | response to interleukin-1 | 5 | 0.6 | 6.7E-02 | 8.7E-01 |
| GO:0032760 | positive regulation of tumor necrosis factor production | 6 | 0.7 | 6.8E-02 | 8.8E-01 |
| GO:0000165 | MAPK cascade | 19 | 2.2 | 6.9E-02 | 8.7E-01 |
| GO:0001843 | neural tube closure | 8 | 0.9 | 6.9E-02 | 8.7E-01 |
| GO:0032869 | cellular response to insulin stimulus | 8 | 0.9 | 6.9E-02 | 8.7E-01 |
| GO:0048280 | vesicle fusion with Golgi apparatus | 3 | 0.3 | 7.7E-02 | 9.0E-01 |
| GO:0045724 | positive regulation of cilium assembly | 3 | 0.3 | 7.7E-02 | 9.0E-01 |
| GO:0015937 | coenzyme A biosynthetic process | 3 | 0.3 | 7.7E-02 | 9.0E-01 |
| GO:1903206 | negative regulation of hydrogen peroxide-induced cell death | 3 | 0.3 | 7.7E-02 | 9.0E-01 |
| GO:0090630 | activation of GTPase activity | 8 | 0.9 | 7.7E-02 | 9.0E-01 |
| GO:0032436 | positive regulation of proteasomal ubiquitin-dependent protein catabolic process | 7 | 0.8 | 7.9E-02 | 9.0E-01 |
| GO:0008285 | negative regulation of cell proliferation | 26 | 3.0 | 8.1E-02 | 9.1E-01 |
| GO:0016569 | covalent chromatin modification | 10 | 1.1 | 8.4E-02 | 9.1E-01 |
| GO:0010506 | regulation of autophagy | 6 | 0.7 | 8.4E-02 | 9.1E-01 |
| GO:0043085 | positive regulation of catalytic activity | 8 | 0.9 | 8.6E-02 | 9.1E-01 |
| GO:0007040 | lysosome organization | 5 | 0.6 | 8.7E-02 | 9.1E-01 |
| GO:0090307 | mitotic spindle assembly | 5 | 0.6 | 8.7E-02 | 9.1E-01 |
| GO:0006468 | protein phosphorylation | 29 | 3.3 | 8.9E-02 | 9.2E-01 |
| GO:0010923 | negative regulation of phosphatase activity | 6 | 0.7 | 9.0E-02 | 9.2E-01 |
| GO:0060173 | limb development | 4 | 0.5 | 9.1E-02 | 9.2E-01 |
| GO:0090161 | Golgi ribbon formation | 3 | 0.3 | 9.1E-02 | 9.2E-01 |
| GO:0031076 | embryonic camera-type eye development | 3 | 0.3 | 9.1E-02 | 9.2E-01 |
| GO:0033197 | response to vitamin E | 3 | 0.3 | 9.1E-02 | 9.2E-01 |
| GO:0010955 | negative regulation of protein processing | 3 | 0.3 | 9.1E-02 | 9.2E-01 |
| GO:0071372 | cellular response to follicle-stimulating hormone stimulus | 3 | 0.3 | 9.1E-02 | 9.2E-01 |
| GO:0014013 | regulation of gliogenesis | 2 | 0.2 | 9.2E-02 | 9.2E-01 |
| GO:2000077 | negative regulation of type B pancreatic cell development | 2 | 0.2 | 9.2E-02 | 9.2E-01 |
| GO:0051563 | smooth endoplasmic reticulum calcium ion homeostasis | 2 | 0.2 | 9.2E-02 | 9.2E-01 |
| GO:0009826 | unidimensional cell growth | 2 | 0.2 | 9.2E-02 | 9.2E-01 |
| GO:0015942 | formate metabolic process | 2 | 0.2 | 9.2E-02 | 9.2E-01 |
| GO:0060545 | positive regulation of necroptotic process | 2 | 0.2 | 9.2E-02 | 9.2E-01 |
| GO:0010825 | positive regulation of centrosome duplication | 2 | 0.2 | 9.2E-02 | 9.2E-01 |
| GO:1903373 | positive regulation of endoplasmic reticulum tubular network organization | 2 | 0.2 | 9.2E-02 | 9.2E-01 |
| GO:2001190 | positive regulation of T cell activation via T cell receptor contact with antigen bound to MHC molecule on antigen presenting cell | 2 | 0.2 | 9.2E-02 | 9.2E-01 |
| GO:0071670 | smooth muscle cell chemotaxis | 2 | 0.2 | 9.2E-02 | 9.2E-01 |
| GO:0050893 | sensory processing | 2 | 0.2 | 9.2E-02 | 9.2E-01 |
| GO:0006461 | protein complex assembly | 10 | 1.1 | 9.5E-02 | 9.2E-01 |
| GO:0051092 | positive regulation of NF-kappaB transcription factor activity | 11 | 1.3 | 9.6E-02 | 9.2E-01 |
| GO:0031175 | neuron projection development | 9 | 1.0 | 9.8E-02 | 9.3E-01 |

The letter E means "10 to the power of." For example, 1.01E+1 means 1.01 * 10^1^ which is 10.1.
